# Pathogen-specific IgE-reactive cytosolic allergenic epitopes of *Aspergillus fumigatus* for immunodiagnostic/immunotherapeutic applications against allergic aspergillosis

**DOI:** 10.1186/s12941-025-00846-z

**Published:** 2026-01-15

**Authors:** Priya Koundal, Sunita Manhas, Shahbaz Aman, Shafiul Haque, Bharat Singh, Shakeel Ahmed Mohammed, Michael Oellerich, Hardeep S. Tuli, Seema Ramniwas, Mehak Dangi, Abdul R. Asif

**Affiliations:** 1 Department of Biochemistry, MM Institute of Medical Sciences and Research, Maharishi Markandeshwar (Deemed to be University), Mullana, Ambala, Haryana 133207 India; 2 Department of Microbiology, Maharishi Markandeshwar Institute of Medical Sciences and Research, Maharishi Markandeshwar (Deemed to be University), Mullana, Ambala, Haryana India; 3 Department of Nursing, College of Nursing and Health Sciences, Jazan University, Jazan, Saudi Arabia; 4 School of Medicine, Universidad Espiritu Santo, 091952 Samborondon, Ecuador; 5 Division of Diagnostics and Biochemistry, Institute of Genomics and Integrative Biology, Mall Road, University Campus, Delhi, 110007 India; 6 Department of Biosciences & Technology and Central Research Cell, MMEC, Maharishi Markandeshwar (Deemed to be University), Mullana, Ambala, Haryana 133207 India; 7 Department of Clinical Chemistry/UMG-Laboratories, University Medical Center, Robert Koch-Str.40, D-37075 Goettingen, Germany; 8 University Centre for Research & Development, University Institute of Pharmaceutical Sciences, Chandigarh University, Gharuan, Mohali, Punjab 140413 India; 9 Centre for Bioinformatics, Maharshi Dayanand University, Rohtak, Haryana India

**Keywords:** Allergens, Antibody, ABPA, Diagnosis, Immunoproteomics

## Abstract

*Aspergillus fumigatus* is a significant fungal pathogen responsible for allergic bronchopulmonary aspergillosis (ABPA), a global respiratory disease characterized by high morbidity and mortality. Accurate diagnosis of ABPA remains challenging due to the overlap of clinical and radiological features with other respiratory infections. This study aimed to identify *A. fumigatus* specific antigens and epitopes to advance immunodiagnostic and immunotherapeutic strategies for ABPA. Clinically confirmed ABPA patients were recruited, and their sera were tested for immunoreactivity against *A. fumigatus* crude and recombinant allergens. Proteomic analysis was done by employing two-dimensional electrophoresis (2DE) immunoblotting of *A. fumigatus* cytosolic fractions using sera from 10 individual patients. IgE-reactive spots identified via Q-TOF mass spectrometry revealed 18 allergenic proteins. Among these, two known allergens (Asp-f12 and Asp-f22) and three predicted allergens (sorbitol/xylulose-reductase, Hsp70-chaperone Hsp88, and Hsp70) exhibited cross-reactivity with allergens from other fungi. Notably, 13 allergenic proteins demonstrated significant immunoreactivity specific to ABPA patient sera. In silico epitope analysis identified 9 *A. fumigatus* specific B-cell and 4 T-cell epitopes with antigenic potential. Molecular docking studies confirmed the binding of representative B-cell and T-cell epitopes to their respective receptors (B-cell receptor and MHC-II complex), demonstrating receptor-specific interactions. These findings highlight pathogen-specific allergens and epitopes that could serve as valuable resources for developing targeted immunodiagnostic and immunotherapeutic tools. This approach may improve the clinical management of ABPA and help reduce its global disease burden.

**Graphical Abstract:**

**Supplementary Information:**

The online version contains supplementary material available at 10.1186/s12941-025-00846-z.

## Highlights

Aspergillosis: Global disease.

Pathogen**-**specific allergen/epitope.

Utility in diagnostics and therapeutics.

**Supplementary Information:**

The online version contains supplementary material available at 10.1186/s12941-025-00846-z.

## Graphical abstract

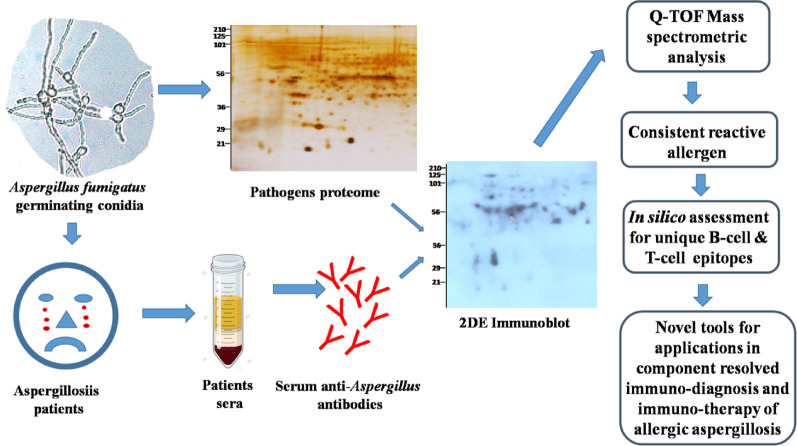

## Introduction

Respiratory distress is strongly associated with air quality, with severe allergic manifestations frequently observed in atopic individuals and patients with chronic obstructive pulmonary disease (COPD), asthma, and emphysema. In such individuals, airborne allergens and fungal pathogens are prime contributors to allergic inflammatory diseases [1–5]. Among these, Allergic Bronchopulmonary Aspergillosis (ABPA) a fungal infection caused by the airborne pathogen *Aspergillus fumigatus* is particularly significant, as it can lead to fatal allergic and invasive infections [6]. In recent years, the prevalence of ABPA in COVID-19 patients has been increasingly reported, raising concerns that demand urgent attention from medical professionals [7–13]. Despite decades of research on Aspergillus induced allergic and invasive infections, effective control of the disease remains elusive due to limitations in diagnostic and treatment regimens [14]. From diagnostic and immunotherapeutic perspectives, researchers have explored conidial, mycelial, and secreted protein fractions of *A. fumigatus* as potential allergens, immunogens, and immune modulators that play critical roles in disease pathogenesis. ABPA patients often exhibit heightened polyclonal responses to multiple allergens and antigens. So, checking the total amount of IgE in the blood as well as the Aspergillus-specific IgE and IgG antibodies has become an important serum-based test for immunodiagnosis [14–16]. To date, a limited number of *A. fumigatus* allergens, including rAsp f1-4 and rAsp f6, have been certified by the World Health Organisation (WHO) and the International Union of Immunological Societies (IUIS). These allergens, produced recombinantly, are utilized in immunodiagnostic platforms such as ImmunoCAP (Phadia, Uppsala, Sweden), but their applications remain limited [4]. Cross-reactivity with homologous fungal allergens has constrained the potential of additional *A. fumigatus* allergens screened for diagnostic use [16].

*A.fumigatus* three-week secretory proteins and cytosolic/germinating conidial proteins are some of the antigen preparations that have been shown to react with ABPA patient sera [15, 16]. Researchers have studied the immunoreactivity of secreted fractions with individual ABPA patient sera, but they have rarely explored the immunoreactivity of cytosolic proteins with individual ABPA patient sera. This gap limits the identification of specific allergens and epitopes for use in immunodiagnostic and immunotherapeutic applications. The point of this study was to find IgE-reactive proteins and the B-cell and T-cell epitopes that are unique to *A. fumigatus* from the cytosolic fraction of the organism. These allergenic proteins and specific antigenic epitopes have a lot of potential for making new ways to diagnose immune problems quickly and accurately, whether they are used alone or together. Furthermore, they could contribute to the advancement of desensitization therapies for allergic ABPA, thus improving disease management and patient outcomes.

## Materials and methods

### Subject recruitment and ethics

Patients clinically diagnosed with respiratory illness were prospectively recruited from the Department of Respiratory Medicine, Maharishi Markandeshwar Institute of Medical Science and Research Centre (MMIMSR), Mullana. The study protocol was reviewed and approved by the Institutional Ethics Committee of MMIMSR. Written informed consent was obtained from all enrolled subjects in accordance with the Declaration of Helsinki and relevant national guidelines [17, 18]. All recruited subjects underwent a detailed clinical evaluation, including a medical history and assessment of respiratory symptoms (shortness of breath, cough, wheezing, and sputum production). Fever or acute illness episodes were documented [19].

### Diagnosis of *A. fumigatus* infection

#### Diagnostic criteria

*Aspergillus* co-infection was diagnosed using revised criteria established by the International Society for Human and Animal Mycology (ISHAM) [19]. Major criteria requirements included: Elevated *A. fumigatus*-specific IgE (≥ 0.35 kUA/L) and total IgE ≥ 500 IU/mL. While at least two of the minor criteria must be included: (i) Specific *A. fumigatus* IgG antibodies (ELISA). (ii) Absolute eosinophil count ≥ 500 cells/µL (normal adult range: 40–400/µL) via automated Coulter counter. (iii) Radiographic evidence of pulmonary opacities/transient infiltrates (ABPA features) [20].

#### Skin Prick test

Skin prick testing was performed on the volar forearm using 0.5 mL of 1:10 diluted A. fumigatus antigen. Negative (buffer saline) and positive (glycerinated histamine acid phosphate) controls were placed at least 2.5 cm from the test site. Readings were taken after 60 min for erythema and wheal formation; a positive test was defined as a wheal diameter ≥ 3 mm [21].

#### Serological analysis

Peripheral blood (5 mL) was collected from all recruited patients and distributed in plain tubes (for serum) and EDTA tubes (for whole blood). Total serum IgE was quantified using commercial sandwich ELISA kits according to the manufacturer’s protocols [19]. Specific IgE (Abbexa Ltd) and IgG (ab247190) against A. fumigatus were measured via indirect ELISA; all procedures followed manufacturer-supplied protocols [22].

### Isolation and identification of *Aspergillus fumigatus*

The *A. fumigatus* strain ITCC-6604, isolated from an Indian ABPA patient, was used as the antigen source for all experimental procedures. In brief, *A. fumigatus* (ITCC-6604) was inoculated onto Sabouraud dextrose agar (SDA) plate and incubated at 37 °C for 1–4 weeks [23]. After observing velvety to powdery bluish-green fungal growth, the isolate was further processed for preliminary identification using a 10% potassium hydroxide (KOH) mount and lactophenol cotton blue (LPCB) staining [24]. After the identification of *A. fumigatus*, the conidia grown on SDA plate were scrapped using sterile scrapper and collected in a 50 ml falcon tubes. Reference strain *A. fumigatus* ATCC 1022 was used for quality control [25].

### Preparation of the cytosolic protein fraction

Crude cytosolic proteins were prepared following a previously described method [16]. Briefly, *A. fumigatus* conidia collected in a falcon tubes were transferred to 200 mL of L-asparagine broth and kept at incubation for 16 h at 37 °C in a shaking incubator (C25KC; New Brunswick Scientific, Edison, USA). After that the germinated conidial biomass was centrifuged at 4 °C, 5000 rpm for 10 min. 5gm of the collected cells were crushed using a mortar and pestle with 0.4 volumes of 0.5 mm glass beads in 10 mM Tris-buffered saline (pH 6.5) for one hour continuously. The resulting slurry was centrifuged at 4 °C, 18,000 rpm for 30 min in a fixed-angle rotor centrifuge (5810R, Eppendorf, Hamburg, Germany). The supernatant, containing the cytosolic proteins, was collected, transferred to fresh tubes, and stored at − 80 °C. Furthermore, protein concentration from fungal conidial cells was determined using the bicinchoninic acid (BCA) assay. BCA standards were prepared (2 mg/mL) for calibration. Samples (2 µL + 23 µL water) and standards were mixed with 200 µL BCA reagent, incubated at 37 °C for 30 min, and read at 562 nm [26, 27].

### Recruitment of patient sera

10 ml Blood sample was collected from clinically diagnosed ABPA patients (*n* = 10) recruited in the current study. Clinical and serological criteria were employed for ABPA diagnosis (Supplementary Table S1a). Blood samples were also collected from healthy subjects as a control (Supplementary Table S1b). All samples were collected after obtaining informed consent and ethical clearance from the Institutional Human Ethics Committee (IHEC) of the Institute of Genomics and Integrative Biology, Delhi, India (approval number: IGIB/IHEC/Jul-2010/13).

### Determination of serum immunoglobulin levels against cytosolic antigens

Modified ELISA methods were used to quantify *Aspergillus*-specific IgE and IgG antibodies in serum samples, as described previously [16]. Total IgE concentrations in ABPA patient sera were measured using a standard kit (IgE ELISA Kit, Catalogue No. E88-108, Bethyl Laboratories, USA). Specific IgG and IgE antibodies against cytosolic *A. fumigatus* antigens were quantified using ELISA [15]. Specific IgG levels against crude *A. fumigatus* antigens were measured with a commercial kit (*Aspergillus fumigatus* specific IgG ELISA Kit, Catalogue No. DEASPG0680, DeMedttec, Germany). IgE antibodies specific to crude and recombinant allergens (rAsp f 1–4 and rAsp f 6) were detected using a robotic ImmunoCAP platform (Thermo Fisher Scientific, USA) according to manufacturer protocols, with results reported in kUA/L.

### Two-dimensional gel electrophoresis, immunoblotting, and identification of matching immunoreactive spots

Cytosolic proteins from 16-hour cultures were precipitated using a 2DE clean-up kit (Catalogue no. 45001737, Cytiva 80648451, GE-Healthcare Biosciences Corporation, Piscataway, NJ). A 40 µg aliquot of purified protein was loaded onto 7 cm IPG strips (pH 4–7, Catalogue No. 1632015, Bio-Rad, USA) and separated. Silver staining of gels was performed following optimized methods [16, 28]. Proteins separated by 2DE were transferred onto PVDF membranes (Amersham Biosciences) using a Mini Protean transfer unit (Catalogue No. 1703930, Bio-Rad, USA). Protein transfer was verified with Ponceau staining, followed by washing. IgE immunoblots were developed using sera from ABPA patients based on previously established protocols [16]. IgE-reactive protein spots on 2DE immunoblots were matched with silver-stained gels using Delta 2-DE software (v4.0, DECODON, Germany). To confirm protein identity, a subset of immunoblots was analyzed directly from PVDF membranes.

### Mass spectrometry-based identification of IgE reactive proteins

Matched spots were manually excised & subjected to in-gel or PVDF-membrane tryptic digestion, followed by peptide separation using C18 reversed-phase nano-LC. Peptides were analyzed using a Q-TOF Ultima Global mass spectrometer (Micromass, UK) equipped with a nano-ESI source. Protein identification was performed using MASCOT searches against SwissProt and NCBInr databases, with stringent confidence thresholds (*p* < 0.05) [15]. Peptide sequences were searched against the NCBI non-redundant protein database using MASCOT (Matrix Science). Functional annotation was performed using UniProt.

### Allergenicity evaluation and epitope prediction

Amino acid sequences of the identified IgE-reactive cytosolic proteins were evaluated for allergenic potential using AllerMatch and the Structural Database of Allergenic Proteins (SDAP). AllerMatch was used to assess sequence similarity against known allergens based on established FAO/WHO criteria, including full-length alignment and contiguous amino acid identity [29]. SDAP was employed to analyze potential allergenicity by comparing physicochemical properties and sequence motifs of the query proteins with curated allergen datasets [30, 31]. Prediction of linear B-cell epitopes was performed using ABCpred and BCEpred servers. ABCpred employs an artificial neural network-based approach with a default window length of 16 amino acids and a threshold score of 0.51 [32], while BCEpred predicts epitopes based on physicochemical properties such as hydrophilicity, flexibility, polarity, and surface accessibility. Only epitopes predicted by more than one algorithm were considered for further analysis [33]. T-cell epitope prediction was carried out using MHCpred [34] and Propred [35], focusing on binding affinity to common HLA class II alleles associated with immune responses in ABPA. MHCpred was used to predict peptide binding affinity based on IC₅₀ values, while Propred was employed to identify promiscuous HLA-DR–binding peptides. Predicted epitopes showing strong or moderate binding affinity across multiple HLA alleles were prioritized.

### Protein–Protein interaction analysis

Functional and biological interactions among the identified IgE-reactive cytosolic allergens were analyzed using the STRING database (version 9.0, http://string-db.org). Protein–protein interaction networks were generated using default parameters, incorporating evidence from experimental data, curated databases, gene co-expression, and text mining. Only interactions with a confidence score ≥ 0.4 were considered significant. The resulting interaction networks were examined to identify functional clusters and to infer potential involvement of allergens in shared biological pathways.

### Identification of unique and non–cross-reactive IgE/IgG epitopes

To minimize potential cross-reactivity, predicted B-cell and T-cell epitopes were subjected to extensive homology searches against allergen and non-allergen databases using BLAST and SDAP-based comparisons. Epitopes showing ≤ 95% sequence identity with known allergens or homologous proteins were considered potentially unique. In addition, property distance (PD) values were calculated, and epitopes with PD values > 10 were classified as non–cross-reactive, as described in previous studies [36–40]. Three-dimensional structures of selected unique epitopes were modelled using iTASSER [41], and structural visualization was performed using UCSF Chimera [42]. Molecular docking studies were conducted using PyRx to evaluate interactions between B-cell epitopes and the Fab domain of the B-cell receptor, as well as between T-cell epitopes and MHC class II molecules. Docking results were assessed based on binding energy scores and interaction stability to prioritize epitopes with favourable receptor-binding characteristics.

## Results

Total10 ABPA patients among asthmatic individuals (Supplementary Table S1a) were recruited in the study. The sera from these patients and healthy controls (Supplementary Table S1b) were assessed for specific IgE and IgG reactivity against crude cytosolic antigens, as well as recombinant allergens of *Aspergillus fumigatus*. Validation of IgG reactivity was performed using a commercial ELISA Kit (Cat No. DEASPG0680, DeMedttec, Germany), and IgE reactivity was evaluated against recombinant *A. fumigatus* allergens (rAsp f 1–4 and rAsp f 6) using an immunodiagnostic setup (ImmunoCAP Allergen Component Testing, USA). In the present study, the three serological approaches evaluated cytosolic crude antigen–based ELISA, commercial crude antigen assay (DeMedttec, DEASPG0680), and ImmunoCAP analysis using recombinant allergens—demonstrated complementary diagnostic performance in differentiating ABPA patients from healthy controls. The cytosolic crude antigen–based ELISA showed high diagnostic sensitivity, as all ABPA patient sera exceeded the established cut-off values for both IgG and IgE, while no reactivity was observed in control sera. This clear separation suggests strong discriminative capacity for initial screening, although the use of crude antigens inherently limits allergen-specific resolution. The commercial crude antigen assay exhibited similarly robust discrimination between ABPA patients and controls, with consistently elevated IgG and IgE levels and relatively lower inter-individual variability compared with the in-house ELISA. This uniformity likely reflects greater assay standardization and supports its reliability in routine diagnostic settings. However, like the cytosolic crude antigen approach, this method primarily reflects overall antigen-specific antibody burden and does not provide component-level diagnostic information. In contrast, ImmunoCAP-based analysis using recombinant allergens offered improved diagnostic specificity through component-resolved profiling. ABPA patients displayed heterogeneous IgE responses to individual allergens (Asp f1, Asp f2, Asp f3, Asp f4, and Asp f6), highlighting variability in sensitization patterns. While this heterogeneity limits the diagnostic sensitivity of any single recombinant allergen, it underscores the value of multi-component panels for enhancing diagnostic accuracy. Collectively, these findings suggest that crude antigen–based assays may be more suitable for sensitive screening, whereas recombinant allergen–based assays provide added specificity and clinical insight. An integrated diagnostic strategy combining both approaches may therefore offer improved accuracy for the diagnosis and characterization of ABPA (Table 1).

**Table 1:** Immunoreactivity of clinical patient and control subject Sera against crude cytosolic and Recombinant allergens of A. fumigatus

S. No. (ABPA Patients)	Cytosolic crude antigen of *Aspergillus fumigatus*		(DeMedttec DEASPG0680)	Recombinant allergens of *Aspergillus fumigatus* (tested on ImmunoCap)						
	Sp. IgG (OD at 492 nm)	Sp. IgE (OD at 410 nm)	Sp. IgG U/ml	Sp. IgE Crude KUA/L	Sp. IgE Asp f1 KUA/L	Sp. IgE Asp f2 KUA/L		Sp. IgE Asp f3 KUA/L	Sp. IgE Asp f4 KUA/L	Sp. IgE Asp f6 KUA/L
	Cut-off 0–0.15		Cut-off 0–12.0	Cut-off 0–0.35						
S1	0.658	1.20	147.12	20.90	45.00	12.70	0.98		38.60	1.90
S2	1.021	0.28	179.12	38.90	5.45	12.20	32.40		45.80	9.73
S3	0.3	0.24	170.38	55.40	8.71	4.42	10.00		1.22	6.87
S4	0.729	0.54	191.89	48.90	30.20	11.50	31.60		3.53	25.60
S5	0.817	0.48	181.34	16.00	25.40	16.20	7.10		25.30	3.94
S6	1.495	1.66	176.46	36.20	60.90	7.38	27.00		24.40	6.94
S7	0.566	0.72	180.32	39.60	18.70	30.30	16.40		1.93	27.90
S8	0.624	0.71	175.86	42.00	52.60	38.10	23.50		27.70	3.18
S9	0.425	0.32	139.89	29.80	6.50	4.31	6.45		0.73	8.16
S10	0.58	0.23	148.53	14.40	2.90	0.48	4.21		1.78	2.27
Controls										
C1	0.00	0.00	9.83	0.05	0.03	0.01	0.00		0.00	0.01
C2	0.00	0.00	2.80	0.29	0.12	0.02	0.01		0.01	0.02
C3	0.00	0.00	10.95	0.07	0.11	0.01	0.00		0.01	0.02
C4	0.00	0.00	21.57	0.04	0.09	0.00	0.00		0.00	0.00
C5	0.00	0.00	4.76	0.04	0.09	0.00	0.00		0.00	0.01

**Fig. 1:**
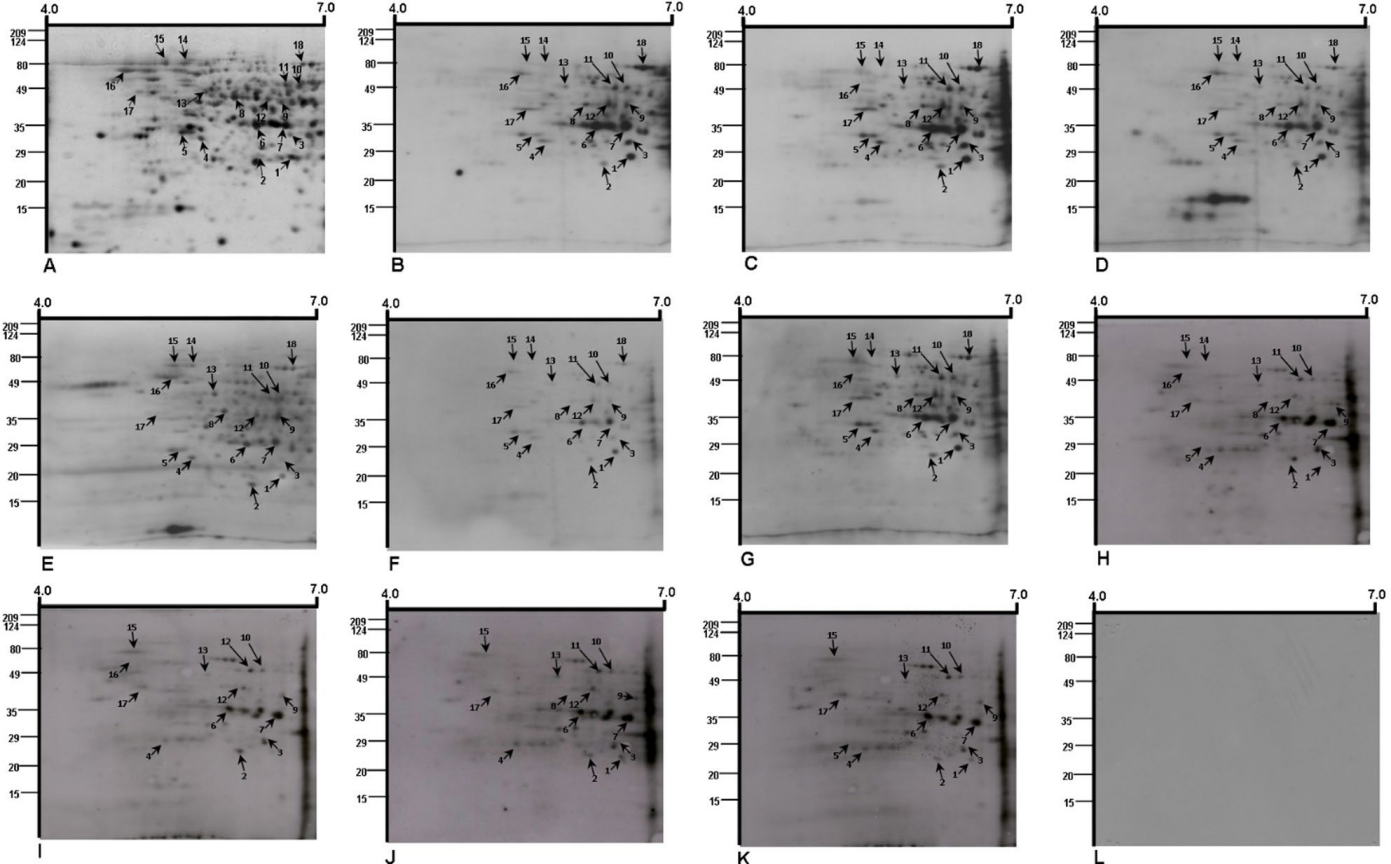
2DE SDS gel and 2DE Western blots of 16 h of the cytosolic protein from the *A. fumigatus* strain ITCC 6604 in a narrow pH range [4–7] developed using hyperreactive immune sera obtained from ABPA patients and healthy control subjects. **A** Silver-stained 2DE SDS gel, **B**–**K** 2DE IgE immunoblot developed with individual sera from ABPA patients and **L** control IgE blot developed with negative pooled sera from healthy individuals

Serum samples were subjected to 2D Western blotting using pooled negative sera from healthy controls as the baseline for control blots. Two-dimensional IgE immunoblots of cytosolic antigens from *A. fumigatus* strain ITCC-6604 were prepared for the 10 individual ABPA patients and compared with silver-stained gels. Representative protein spots were marked and sliced from 2DE gels for further analysis (Fig. 1A). Immunoblots showed erratic IgE-reactive profiles (Fig. 1B–K).

### Identification of allergenic proteins

Protein spots from silver-stained 2DE gels were manually excised, digested and analyzed using Q-TOF MS/MS. MASCOT searches identified 18 allergenic proteins of *A. fumigatus* (Supplementary Table S4). Six web-based tools confirmed the allergenic potential of these proteins. Among these proteins, two known allergens, Asp-f 22 (Spot No. 8) and Asp-f 12 (Spot No. 15), were identified, along with three predicted allergens: sorbitol/xylulose-reductase Sou1-like (Spot No. 1), molecular chaperone Hsp70 (Spot No. 14), and Hsp88 (Spot No. 16). These five proteins exhibited significant sequence homology with other allergen sources, indicating potential cross-reactivity. Thirteen proteins displayed no significant homology with allergens in the databases (allermatch and SDAP). Eleven of these proteins, consistently showing IgE reactivity with at least 8 out of 10 ABPA patients and hence these were marked as potential cytosolic allergens of *A. fumigatus* (Table 2). Seven of the identified proteins were linked to carbohydrate metabolism (Supplementary Information: Fig. S1).

**Table 2:** In Silico assessment of all identified IgE reactive proteins.

Spot No.	Protein name	Biological function	Number of positive reactivity with ABPA patients	Allergenicity prediction (Sequence homology with other allergens)
				**Allermatch & SDAP**
1	Sorbitol/xylulose reductase Sou1-like	Oxidoreductase	8	Cla h 8, *Cladosporium herbarum*, (75%) Alt a 8, *Alternaria alternate*, (73%)
2*	Proteasome component Pre9	Hydrolase, Threonine protease	10	No significant sequence matches with other fungal allergens
3*	Pyridoxine biosynthesis protein	Catalytic activity	8	No significant sequence matches with other fungal allergens
4*	Spermidine synthase	Transferase	10	No significant sequence matches with other fungal allergens
5	Inorganic diphosphatase	Hydrolase	7	No significant cross reactivity with other fungal allergens
6*	Fructose-bisphosphate aldolase, class II	Lyase	10	No significant sequence matches with other fungal allergens
7*	Transaldolase	Transferase	10	No significant cross reactivity with other fungal allergens
8	Enolase/Asp f 22	Lyase	6	Pen c 22.0101, *Penicillium citrinum*, (94%) Alt a 5, *Alternaria alternata*,* (88%)* Cla h 6, *Cladosporium herbarum*,* (86%)* Cur l 2.0101, *Curvularia lunata*, (81%)
9*	Adenosyl homocysteinase	Hydrolase	10	No significant sequence matches with other fungal allergens
10*	Glucose-6 phosphate isomerase	Isomerase	10	No significant sequence matches with other fungal allergens
11*	ATP citrate lyase subunit (Acl), putatibe	Lyase, Ligase	10	No significant cross reactivity with other fungal allergens
12*	6 phosphogluconate dehydrogenase Gnd1	Oxidoreductase, NADP binding	10	No significant sequence matches with other fungal allergens
13*	Secretory pathway gdp dissociation inhibitor	Rab-GDP dissociation	9	No significant sequence matches with other fungal allergens
14	Hsp70 chaperone Hsp88	ATP binding	2	Mala s 10, *Malassezia sympodialis*,* (51%)* Cla h 5.0101, *Cladosporium herbarum*,* (28%)*
15	Asp f12/Heat shock protein P90/Hsp90	ATP/unfolded protein binding	6	Cor a 1, *Corylusavellana* (57%), Man e 5, *Manihotesculenta* (55%) Hev b 5, *Heveabrasiliensis* (54%)
16	Molecular chaperone Hsp70	ATP binding	8	Cla h 5.0101, *Cladosporium herbarum*,* (84%)* Pen c 19, *Penicillium citrinum*,* (72%)* Cor a 10, *Corylus avellana*, (61%) Mala s 10, *Malassezia sympodialis*,* (31%)*
17*	ATP synthase F1, beta subunit	Hydrolase, ATP/metal ion binding	10	No significant sequence matches with other fungal allergens
18	Mitochondrial aconitate hydratase	Tricarboxlic acid cycle, Fe/S ion binding	6	No significant match with any known allergen in SDAP

Consistent reactive non-cross-reactive proteins are marked with (*) in the table

### Epitope prediction and allergenicity assessment

Amino acid sequences of the identified proteins were analyzed using ABCpred, BCEpred, MHCpred, and Propred to predict B-cell and T-cell binding epitopes. In silico protein–protein interaction analyses were conducted using String 9.0. Detailed analyses identified four unique T-cell epitopes (National Patent filed, Ref-E-1/44383/2024-DEL) and nine unique B-cell epitopes (National Patent filed, Ref-E-1/23616/2024-DEL) with no homology to fungal allergens and significant PD values. The T-cell epitope (GKEIKVETV) from the ATP citrate lyase subunit protein was highlighted for its lack of allergen matches and low sequence similarity with other fungal species. Three other T-cell epitopes Pyridoxine biosynthesis protein (AIVQAVTHY), Glucose-6-phosphate isomerase (DFSKNFLTE), and ATP citrate lyase subunit (YYININSVR) also showed no fungal allergen matches (Supplementary Information: Table S3). Nine B-cell epitopes were further modelled using iTASSER for docking studies. The epitopes demonstrated variable PD values (0–13). Among them, the KYPEMLKGCYGVSEETTTGV epitope of adenosyl homocysteinase showed significant homology with *Verticillium longisporum* and *Cudoniella acicularis* (Supplementary Information: Table S2).

### Docking studies

**Fig. 2:**
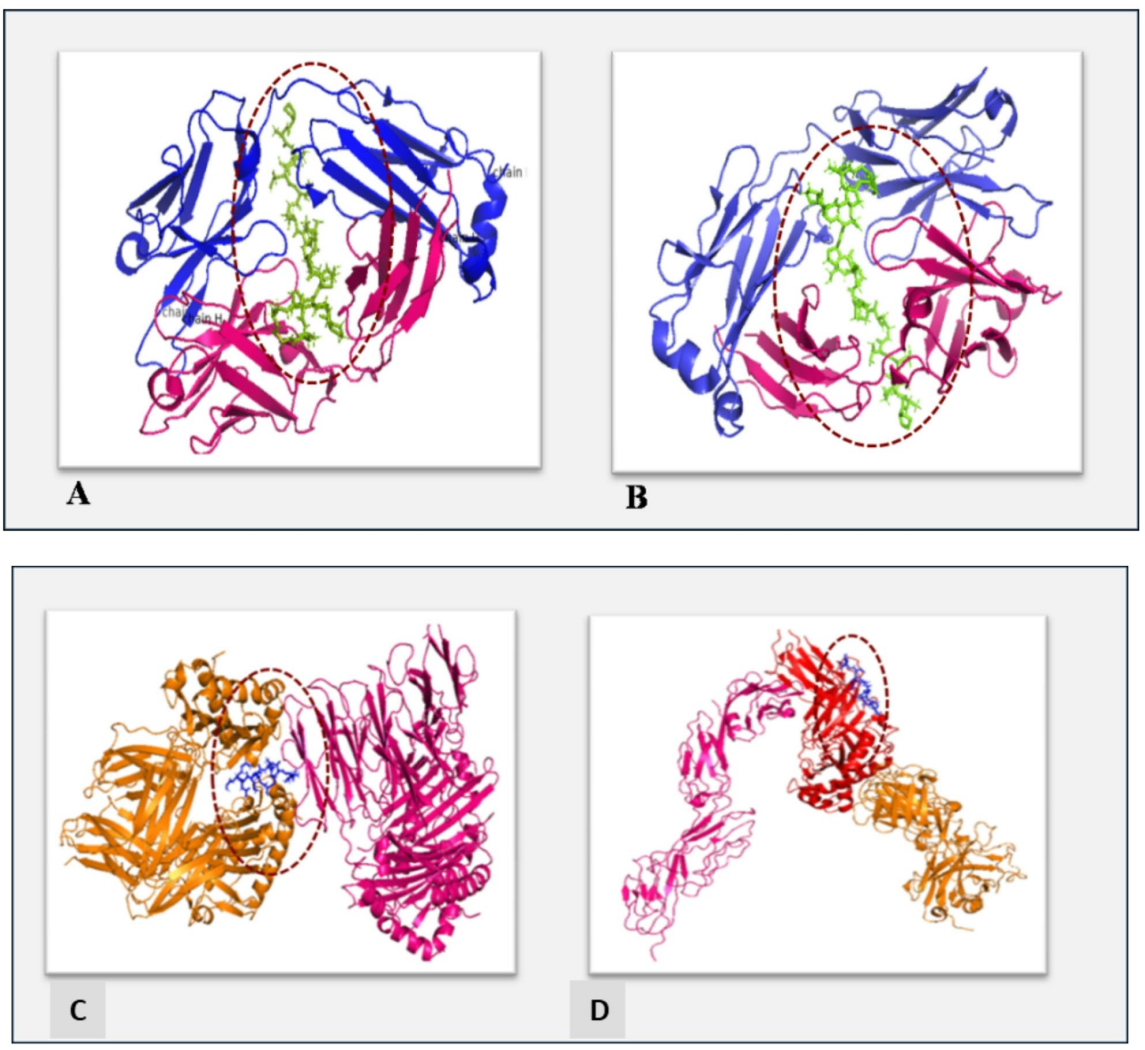
In silico modeling of B-cell and T-cell epitope–receptor interactions. **A** Interaction between the B-cell epitope derived from fructose-bisphosphate aldolase class II and the Fab domain of the B-cell receptor (BCR; PDB ID: 5IFH). **B** Interaction between the B-cell epitope of the ATP synthase F1 beta subunit and the Fab domain of the BCR. The BCR light chain is shown in purple and the heavy chain in pink. **C** Interaction between the predicted T-cell epitope (ligand) from ATP citrate lyase subunit (Acl) and HLA-DRB1. **D** Ternary complex showing the T-cell receptor (red), peptide–MHC II complex (pink), and CD4 co-receptor (orange). In all panels, epitope binding sites are highlighted with dotted circles

Molecular docking revealed favorable binding interactions between B-cell epitopes and the antigen-binding fragment (Fab) of the BCR (ID: 5IFH). The epitopes from fructose-bisphosphate aldolase (EYAQEKNFAIPAVNVTSSST) and ATP synthase F1 protein (PARDTGAPIKIPVGPGTLGR) exhibited binding energies of −13.8 kcal/mol (Fig. 2A) and − 13.5 kcal/mol (Fig. 2B), respectively (Table 3). For T-cell epitopes, docking studies were performed with MHC II allele HLA-DRB1*01:01 (ID: 1AQD) and the human ternary complex (ID: 3T0E). The ATP citrate lyase subunit epitope showed binding energies of −14.1 kcal/mol and − 12.8 kcal/mol, respectively (Fig. 2C, D).

**Table 3:** Binding energies of selected B-cell and T-cell epitopes of IgE-reactive A. fumigatus proteins with BCR and HLA-DRB1, respectively.

Proteins	B-cell epitope	Binding energy with BCR (kcal/mol)
Spermidine synthase	ATRNVREPVRTWSREEEERL	−7.5
Fructose-bisphosphatealdolase, class II*	EYAQEKNFAIPAVNVTSSST	−13.8
Adenosylhomocysteinase	GKKYVEFGTTGKKPVGVYVL	−8.0
	AWKGETEEEYQWCLEQQLNA	−9.2
Glucose-6 phosphate isomerase	EWKGYTGKKITTIINIGIGG	−10.6
ATP citrate lyase subunit (Acl), putative	EWKGYTGKKITTIINIGIGG	−10.6
Secretory pathway gdp dissociation inhibitor	QIEEKFFGPPIPLYEPLDSG	−8.9
	SHFETTTDDVRDLYKRATGE	−10.7
ATP synthase F1, beta subunit*	PARDTGAPIKIPVGPGTLGR	−13.5

Protein	T-cell epitope	Binding energy (Kcal/mol)
ATP citrate lyase subunit (Acl), putative	GKEIKVETV	With HLA- DRB1-14.1
		−12.8
Pyridoxine biosynthesis protein	AIVQAVTHY	–9.44
Glucose-6-phosphate isomerase	DFSKNFLTE	–8.71
ATP citrate lyase subunit	YYININSVR	–10.07

The B-cell epitopes of proteins marked with (*) were selected for Docking studies with receptors

### Cross-reactivity

Cross-reactivity assessments showed that most epitopes with high sequence identity matched fungal allergens, except for select B-cell and T-cell epitopes that demonstrated unique and non-cross-reactive properties. The predicted B-cell epitopes identified across multiple Aspergillus fumigatus proteins showed low property distance (PD) values and high sequence conservation with known fungal allergens, indicating a potential for IgE-mediated cross-reactivity. Several epitopes originating from the proteasome component Pre9, pyridoxine biosynthesis protein, spermidine synthase, fructose-bisphosphate aldolase class II, and transaldolase displayed PD values mainly ranging from 8.9 to 12.0 when compared with allergens from clinically relevant fungi such as Cladosporium, Penicillium, Malassezia, Alternaria, Candida, and Fusarium. Homology analysis further revealed a high degree of sequence conservation, with most epitopes sharing ≥ 95% and often 100% identity across diverse fungal species. Although a subset of epitopes did not show direct matches with known allergens, their strong sequence conservation suggests potential immunological relevance (Table S2). Similarly, the predicted T-cell epitopes derived from multiple A. fumigatus proteins exhibited low PD values relative to established fungal allergens, pointing toward possible T-cell–mediated cross-reactivity. Epitopes from the same key proteins showed consistently low PD values, including instances of PD = 0.00, reflecting near-identical physicochemical properties to allergens from Cladosporium, Penicillium, Alternaria, Malassezia, and Fusarium species. BLASTP analysis demonstrated high sequence conservation, predominantly with 100% identity across different fungal taxa. Even for epitopes lacking identifiable allergen counterparts, the marked sequence conservation supports their potential immunological significance (Table S3). These findings highlight the potential of these epitopes for specific immunodiagnostics and therapeutic applications.

## Discussion

The spectrum of ABPA has appeared to be vast; in the past few decades, it has become a major health concern among patients suffering from respiratory illness. During the COVID-19 pandemic, several patients were diagnosed with aspergillosis as a secondary infection [1–3, 8, 10, 11, 13]. The diagnosis of ABPA relies mainly on Rosenberg–Patterson or modified ISHAM’s major & minor criteria, which includes elevated *A. fumigatus*-specific IgE as one of the major criteria [20]. This specific IgE are mainly reactive against crude antigens, which are highly sensitive but less specific due to cross-reactivity with other fungal allergens [43]. Some WHO/IUIS-certified allergens of *A. fumigatus* have been identified mainly from secreted fractions. Among them recombinant allergens including Asp f 1, 2, 3, 4 and Asp f 6 are widely used for diagnostic purpose, but their utility has been challenged several times due to a lack of discrimination power [44–47]. When the conidia of the inhaled *A. fumigatus* germinate, the released cytosolic proteins directly exposed to the lung microenvironment and immune cells. These early-released cytosolic proteins can trigger initial allergic responses and contribute to the development of ABPA [48]. However, cytosolic allergens have not been thoroughly characterized in the literature. Therefore, identifying and characterizing allergenic proteins from the cytosolic fraction is essential for developing newer diagnostics or immunotherapeutic for ABPA or aspergillosis. Hence, we conducted the present study, which involved an ELISA-based analysis of the immunoreactivity of ABPA patients and control subjects to crude cytosolic and recombinant *A. fumigatus* allergens (rAsp f 1–4 and rAsp f 6) to select the immune sera of true-positive ABPA patients for further use in identification of IgE-reactive cytosolic allergens of *A. fumigatus*. All the serum samples in study group were found positive with a commercial IgG immunoassay kit against & recombinant allergens (rAsp f 1–4 and rAsp f 6) on ImmunoCap. The *A. fumigatus* strain ITCC-6604, isolated from ABPA patient were cultured on SDA and the fungal conidia were further subjected for protein extraction. After that 2DE Western blotting of the crude cytosolic fraction of *A. fumigatus* identified 18 proteins with significant MASCOT scores (84–830) and sequence coverage of 7–40%. Out of total 18 allergen proteins, 9 of them were reactive with sera of all ABPA patients (n-10), while protein identified on spot 13 were reactive with sera of 9 patients & protein on spot 3 were reactive with sera of 8 patients. Notably, none of these 11 proteins showed significant sequence similarity or cross-reactivity with previously reported allergens, based on whole amino-acid–sequence analysis using in silico allergenicity tools. Previous studies also reported the use of recombinant allergens (rAsp f 1–4 and rAsp f 6) for the immune-diagnosis purpose. These allergens are secreted and released after the invasion and colonization of the *A. fumigatus* in human host. It was already shown that such secreted allergens have high IgE and IgG reactivity but their cross-reactivity presents major drawback [15]. Similar to other studies, current study also established the superior competence of recombinant allergens of *A. fumigatus* such as rAsp f 1–4 and rAspf 6 in comparison to crude antigens. Whereas rAsp f 1 and rAsp f 2 were found to be the best in discriminating between ABPA and fungal sensitization, combinations of 5 recombinant ABPA (rAsp f 1–4 and rAsp f 6) and specific IgE have been recommended for clearer discrimination of different types of *Aspergillus*-sensitized patients [44–47]. In silico assessments of each protein via allergen databases (allermatch and SDAP) revealed that 2 known (Asp f 12 and Asp f 22) and 3 predicted (Sou1, molecular chaperone Hsp70 chaperone-Hsp88 and Hsp70) allergens of *A. fumigatus* [49] significantly matched with other fungal allergens (Table 3). The allergen Asp f 22 was associated with 4 fungal allergens, Pen-c22.0101, *Penicillium citrinum* (94%), Alt-a5 *Alternaria alternata* (88%), Cla-h6 *Cladosporium herbarum* (86%) and Cur-l2.0101, *Curvularia lunata* (81%). The allergen Asp f 12 shares significant sequence homology with three potential allergens that were named as Cor-a1, *Corylus avellana* (57%), Man-e5, *Manihot esculenta* (55%), and Hev-b5, *Hevea brasiliensis* (54%). Among the 3 identified predicted allergens of the *A. fumigatus* molecular chaperone Hsp70, Cla-h5.0101 *C. herbarum* (84%) and Pen-c19 *P. citrinum* (72%) shared sequence homology with 2 fungal allergens, whereas the Hsp70 chaperone-Hsp88 contained a 51% homologous amino acid sequence with the yeast allergen Mala-s10 *Malassezia sympodialis.* The protein of spot No. 1 (Sou1) has 78% and 73% sequence homology with Cla-h8, *C. herbarum*, and Alt-a8, *A. aalternata* allergens, respectively. Predictive analysis of these immunodominant proteins was carried out to determine their biological interactions and to elucidate their role in different metabolic/cellular processes. The biological functions of the 7 most closely related genes (spot nos. 5–8, 10, 12, and 18) among the 18 identified proteins involved in carbohydrate metabolism were validated by the String server version 9.0 (Supplementary Information: Fig S1). However, a detailed analysis of the 11*-marked IgE reactive proteins in Table 3 (consistently IgE reactive) might reveal their putative roles as host pathogenic determinants that may be involved in the development of ABPA.

It is important to note that any protein claims an antigen by the presence of antigenic epitopes, which may consist of a few amino acids. Hence, only 1% of the total protein sequences could be responsible for potential antigenicity. Therefore, we performed a BLAST analysis of the sequence similarity of the epitopes against allergens of fungal microbial pathogens and attempted to identify *A. fumigatus*-specific antigenic epitopes that did not match any fungal allergen. Successfully, nine B-cell antigenic epitopes were identified from the proteins (ATP citrate lyase subunit (Acl), spermidine synthase, fructose-bisphosphate aldolase, class II, adenosy lhomocysteinase, 6 phosphogluconate dehydrogenase Gnd1 and ATP synthase F1, beta subunit) (Supplementary Table S2). However, four *A. fumigatus* specific T-cell epitopes were associated with the proteins ATP citrate lyase subunit (Acl), putative pyridoxine biosynthesis protein, and glucose-6 phosphate isomerase, which did not match with any other reported fungal allergens (Supplementary Table S3). To prove the potency of the predicted epitopes, docking studies of representative B-cell epitopes (ligands) have been performed with variable regions in the antigen-binding fragment (Fab) of the BCR (ID: 5IFH) [50], and docking studies of T-cell epitopes have also been performed with the human ternary complex (T-cell receptor, peptide-MHC II molecule, and CD4 receptor) and the MHC II allele HLA-DRB1* 01: 0. The efficiency of ligand and receptor binding has been reflected in terms of binding energies, which are quite significant according to standard parameters. These epitopes, as well as the full-length protein, are important because of their immunodiagnostic and immunotherapeutic utility. However, a study identified potential biomarkers for diagnosing ABPA by profiling the biotinylated surface proteome of *A. fumigatus* that could be found to be more equally promising than immuno-proteomes [51].

## Conclusions

The present study was undertaken to identify IgE-reactive allergens of *Aspergillus fumigatus* and to characterize their associated B-cell and T-cell epitopes with potential relevance for early detection and improved management of ABPA. Clinically well-defined ABPA patients were carefully screened using established diagnostic criteria, including total IgE, specific IgG and IgE against crude *A. fumigatus* antigens, and specific IgE against recombinant allergens. Immunoproteomic analysis using two-dimensional electrophoresis and immunoblotting revealed 18 IgE-reactive cytosolic proteins, of which 11 showed consistent reactivity across patient sera. The specificity of these allergens and their limited cross-reactivity were supported by comparative sequence analysis against multiple allergen databases. Subsequent in silico analyses identified nine B-cell epitopes and four unique T-cell epitopes with favourable antigenicity, binding affinity, and structural compatibility with immune receptors. Protein–protein interaction analysis further suggested that several of these allergens are co-expressed and predominantly involved in carbohydrate metabolism, underscoring their biological relevance during fungal growth and host interaction. Collectively, these findings enabled the identification of a focused panel of 11 IgE-reactive cytosolic proteins, including six in silico-validated candidates with well-defined epitope profiles.

Although experimental validation of the predicted epitopes remains necessary, the identified allergens and their associated epitopes represent promising candidates for the development of more specific, multiplexed diagnostic platforms, such as peptide- or protein-based arrays, that may facilitate earlier and more accurate differentiation of ABPA from fungal sensitization. In addition, the consistent IgE-reactive proteins and their B-cell epitopes provide a rational foundation for future peptide-based immunotherapeutic approaches, including targeted desensitization strategies. Overall, this study expands the current understanding of the cytosolic allergen repertoire of *A. fumigatus* and provides a valuable framework for future translational studies aimed at improving the diagnosis and treatment of ABPA.

## Supplementary Information

Supplementary Material 1.

## Data Availability

The data presented in this study are available upon reasonable request from the corresponding author.
