# Supplementary material for: Pathogen-specific IgE-reactive cytosolic allergenic epitopes of Aspergillus fumigatus for immunodiagnostic/immunotherapeutic applications against allergic aspergillosis
Source: Ann Clin Microbiol Antimicrob. 2026 Jan 15;25:7. doi: 10.1186/s12941-025-00846-z (PMC12882445; doi:10.1186/s12941-025-00846-z)
Supplement: Supplementary file 1 — Supplementary Material 1. [file 12941_2025_846_MOESM1_ESM.docx]

**SUPPLEMENTARY INFORMATION**

Pathogen-specific IgE-reactive cytosolic allergenic epitopes of *Aspergillus fumigatus* for immunodiagnostic/immunotherapeutic applications against allergic aspergillosis

**Figure S1:** Schematic representation of the protein‒protein interactions of the 18 identified IgE-reactive proteins generated by the web-based software String 9.0.

**Figure S2:** 2DE SDS gel and 2DE Western blots of 16 h of the cytosolic protein from the *A. fumigatus* strain ITCC 6604 in a narrow pH range (4-7) developed using hyperreactive immune sera obtained from ABPA patients and healthy control subjects. (A) Silver-stained 2DE SDS gel, (B-K) 2DE IgE immunoblot developed with individual sera from ABPA patients and (L) control IgE blot developed with negative pooled sera from healthy individuals.

**Table S1a:** Clinical details of the ABPA patients included in the study.

**Table S1b:** Clinical details of healthy individual (control) used in the study.

**Table S2:** B-cell epitopes of proteins showing property distance (PD) values and homology search results.

**Table S3:** T-cell epitopes of proteins with binding distance values with fungal *allergens.*

**Table S4:** Detailed data from Q-TOF MS/MS analysis of identified IgE-reactive proteins of *Aspergillus fumigatus*.

**
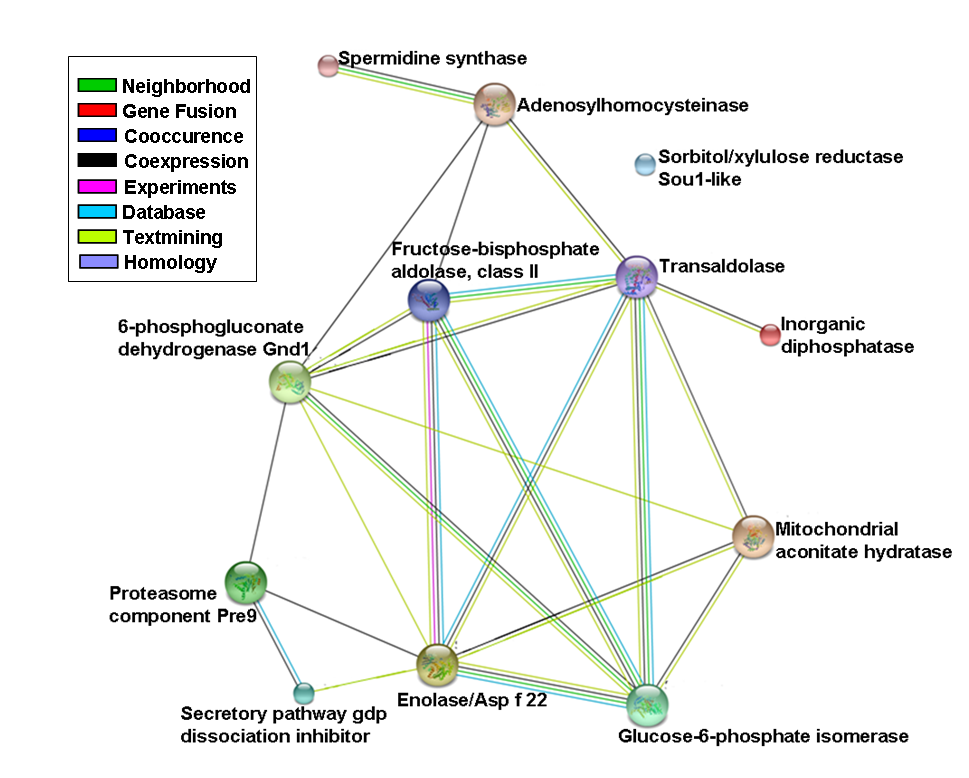
**

**Fig. S1:** Schematic presentation of the protein-protein interaction of the all 18 identified IgE reactive proteins, generated by the web-based software String 9.0.

| **Table S1a:** Clinical details of ABPA patients used in the study. | | | | | | | |
| --- | --- | --- | --- | --- | --- | --- | --- |
| **Patients S. No.** | **Age/Sex** | **X-ray impression** | **Clinical**  **symptoms** | **Skin prick test** | **Sp. IgG**  **(OD at 492 nm)** | **Sp. IgE**  **(OD at 410 nm)** | **Total IgE**  **(IU/mL)** |
| **S1** | 32/F | Infiltrative opacities in right upper zone and mucus plugging | Cough - Yes  Breathlessnes -Yes  Wheezing - No  Sputum - No  FEV1- 40% | Wheal size  (10 mm) | 0.473 | 0.403 | 958.3 |
| **S2** | 25/F | Evidence of bronchiectasis in bilateral upper zones | Cough - No  Breathlessnes - Yes  Wheezing - No  Sputum - No  FEV1- 61% | Wheal size  (10 mm) | 0.522 | 0.224 | 1333.0 |
| **S3** | 67/F | Infiltrative opacities in left lower zone | Cough - Yes  Breathlessnes -Yes  Wheezing - Yes  Sputum - Yes  FEV1- 38% | Wheal size  (9 mm) | 0.706 | 0.305 | 1312.5 |
| **S4** | 23/M | Non-homogenous opacities in right lower zone | Cough - Yes  Breathlessnes -Yes  Wheezing - Yes  Sputum - No  FEV1 - 90% | Wheal size  (8 mm) | 0.602 | 0.307 | 562.5 |
| **S5** | 40/F | Mucus plugging | Cough - Yes  Breathlessnes - Yes  Wheezing - Yes  Sputum - No  FEV1- 54% | Wheal size  (8 mm) | 0.510 | 0.182 | 550.0 |
| **S6** | 26/M | Bronchiectatic changes in right lower zone | Cough - Yes  Breathlessnes - Yes  Wheezing - Yes  Sputum - No  FEV1 - 58% | Wheal size  (9 mm) | 0.407 | 0.234 | 520.8 |
| **S7** | 58/M | Few noduloinfiltrative opacities in left upper zone | Cough - Yes  Breathlessnes - Yes  Wheezing - No  Sputum - Yes  FEV1- 64% | Wheal size  (10 mm) | 0.712 | 0.300 | 937.5 |
| **S8** | 40/F | Infiltrative opacities in bilateral upper zones | Cough - Yes  Breathlessnes -Yes  Wheezing - No  Sputum - Yes  FEV1 - 38% | Wheal size  (10 mm) | 0.721 | 0.178 | 413.3 |
| **S9** | 43/F | Infiltrative opacities in right upper and mid zone | Cough -Yes  Breathlessnes-No  Wheezing -No  Sputum-Yes  FEV1- 77% | Wheal size  (10 mm) | 0.588 | 0.517 | 2021 |
| **S10** | 19/M | Bronchiectatic changes in bilateral lower zones | Cough -No  Breathlessnes-Yes  Wheezing -No  Sputum-No  FEV1- 59% | Wheal size  (13 mm) | 0.708 | 0.608 | 2333 |

| **Table S1b:** Clinical details of healthy individual (control) used in the study. | | | | | | | |
| --- | --- | --- | --- | --- | --- | --- | --- |
| **Patients S. No.** | **Age/Sex** | **X-ray impression** | **Clinical**  **symptoms** | **Skin prick test** | **Sp. IgG**  **(OD at 492 nm)** | **Sp. IgE**  **(OD at 410 nm)** | **Total IgE**  **(IU/mL)** |
| C1 | 25/M | Clear lungs with a clearly outlined chest cavity | Cough - No  Breathlessnes -No  Wheezing - No  Sputum - No  FEV1- 90% | No Induration | 0.0 | 0.0 | 0.0 |
| C2 | 37/M | Clear lungs with a clearly outlined chest cavity | Cough - No  Breathlessnes -No  Wheezing - No  Sputum - No  FEV1- 90% | No Induration | 0.0 | 0.0 | 0.0 |
| C3 | 53/M | Clear lungs with a clearly outlined chest cavity | Cough - No  Breathlessnes -No  Wheezing - No  Sputum - No  FEV1- 90% | No Induration | 0.0 | 0.0 | 0.0 |
| C4 | 28/F | Clear lungs with a clearly outlined chest cavity | Cough - No  Breathlessnes -No  Wheezing - No  Sputum - No  FEV1- 95% | No Induration | 0.0 | 0.0 | 0.0 |
| C5 | 39/F | Clear lungs with a clearly outlined chest cavity | Cough - No  Breathlessnes -No  Wheezing - No  Sputum - No  FEV1- 80% | No Induration | 0.0 | 0.0 | 0.0 |

**Table S2:** B-cell epitopes of proteins showing property distance (PD) values and homology search results.

| **Protein** | **B cell epitope** | **PD value with a fungal allergen** | **blastP results** |
| --- | --- | --- | --- |
| Proteasome component Pre9 | CDLKQGYTQHGGLRPFGVSF (115-134) | 11.4 *Cladosporiu, herbarum* | *Monosporascus sp. 5C6A* 100% |
|  | EFQLYQSNPSGNYGGWKATS (144-163) | 10.5 *Malassezia sympodialis* | ***Thelocarpon* *superellum* 100%** |
|  | GKTKEGKIYHHLWNADEINA (216-235) | 11.09 *Rhodorula mucilaginosa* | ***Penicilliopsis zonata* 100%** |
| Pyridoxine biosynthesis protein | GSGIFKSGDAKKRAKAIVQA (249-268) | 10.7 *Coprinus comatus* | *Penicillium rolfsi*100% |
|  | MASNGTNGASASNSFTVKAG (1-19) | 8.9 *Malassezia sympodialis* | ***Monascus* *ruber* 81%** |
|  | EGAAMIRTKGEAGTGDVVEA(149-168) | 10.7 *Cladosporium herbarum* | ***Scytalidium* *lignicola* 100%** |
|  | EKGRLPVVNFAAGGVATPAD(215-234) | 11.7 *Cladosporium herbarum* | ***Trichophyton* *violaceum100%*** |
| Spermidine synthase | ATRNVREPVRTWSREEEERL (249-268) | No allergen | ***Penicillium* *lagena 95%*** |
|  | PVAEYAYTTIPTYPSGQIGF (233-252) | 11.0 *Penicillium citrinum* | ***Kickxella* *alabastrina* 100%** |
|  | GGGDGGVLREVVKHESVEEA (94-113) | 10.4 *Penicillium citrinum* | ***Pseudocercospora* *eumusae* 100%** |
|  | IITDSSDPEGPAESLFQKPY (163-182) | 11.0 *Cladosporium herbarum* | ***Teloschistes* *flavicans* 100%** |
| Fructose-bisphosphate aldolase, class II | EIGITGGEEDGVNNEDVDNN (175-194) | 11.59 *Cladosporium herbarum* | ***Verticillium* *longisporum***100% |
|  | SAVGNPDGEDKPNKKYFDPR (314-333) | 11.06 *Cladosporium herbarum* | *Trichophyton tonsurans* 100% |
|  | AAGFGNVHGVYKPGNVRLHP (220-239) | 10.98 *Penicillium citrinum* | ***Purpureocillium* *lavendulum* 100%** |
|  | EYAQEKNFAIPAVNVTSSST (24-43) | 10.6 *Coprinus comatus* | *Talaromyces islandicus* 95% |
| Transaldolase | AAVQKGKKEGKTLDEQVDAT (55-74) | 4.23 *Penicillium chrysogenum* | ***Paecilomyces variotii* 85%** |
|  | GDFATIGKYKPQDATTNPSL (20-39) | 0 *Cladosporium cladosporioides* | *Hortaea werneckii* 100% |
|  | EIIPGKVSTEVDARFSFDTQ(88-107) | 1.42 *Penicillium chrysogenum* | ***Cirrosporium* *novae-zelandiae 95%*** |
| Adenosyl homocysteinase | GKKYVEFGTTGKKPVGVYVL (384-403) | 10.8 *Candida albicans* | ***Penicillium lagena 95%*** |
|  | AWKGETEEEYQWCLEQQLNA (102-121) | No allergen | ***Penicillium salamii 95%*** |
|  | KYPEMLKGCYGVSEETTTGV (145-164) | No allergen | ***Cudoniella acicularis* 100%** |
|  | KANAKSVQNIKPQVDRYLMP (315-334) | 10.0 *Cladosporium herbarum* | ***Pseudovirgaria hyperparasitica*100%** |
| Glucose-6 phosphate isomerase | IQKELETPGAGGDHDASTSG (522-541) | 9.8 *Malassezia sympodialis* | ***Rasamsonia emersonii* 100%** |
|  | EWKGYTGKKITTIINIGIGG (145-164) | 11.63 *Curvularia lunata* | ***Thelocarpon superellum 95%*** |
|  | ASKTFTTAETTTNANSAKKW (214-223) | 10.4 *Schizophyllum commune* | *Talaromyces pinophilus* 100% |
|  | ESNGKAITRTGEYVKYTTGP (363-382) | 11.1 *Alternaria alternata* | ***Macrophomina phaseolina* 100%** |
|  | LSTNEEEVTKFGIDKKNMFG (251-270) | 10.6 *Penicillium citrinum* | ***Aureobasidium pullulans* 100%** |
| ATP citrate lyase subunit (Acl), putatibe | IQKELETPGAGGDHDASTSG (522-541) | 9.8 *Malassezia sympodialis* | ***Rasamsonia emersonii*100%** |
|  | ASKTFTTAETTTNANSAKKW (214-233) | 10.6 *Alternaria alternata* | *Talaromyces pinophilus* 100% |
|  | ESNGKAITRTGEYVKYTTGP (363-382) | 11.1 *Alternaria alternata* | ***Macrophomina phaseolina* 100%** |
|  | LSTNEEEVTKFGIDKKNMFG (251-270) | 10.3 *Cladosporium cladosporioides* | ***Aureobasidium pullulans* 100%** |
| 6 phosphogluconate dehydrogenase Gnd1 | GGEEGARYGPSLMPGGNEEA (150-169) | 10.1 *Fusarium culmorum* | ***Erysiphe* *pulchra*100%** |
|  | EEGDIIIDGGNSHFPDSNRR (113-132) | 11 *Cladosporium herbarum* | ***Ogataea* *philodendra* 100%** |
|  | AAKSDGEACCDWVGDEGAGH(181-200) | 11.14 *Candida albicans* | ***Verticillium* *longisporum***100% |
|  | WRNVVSKGALWGIPTPAFST (242-443) | 11.2 *Malassezia furfur* | ***Lasiodiplodia* *theobromae* 100%** |
| Secretory pathway gdp dissociation inhibitor | YGNVRPGEEPWKKYGRVNDW (58-77) | 12.04 *Cladosporium cladosporioides* | ***Talaromyces* *pinophilus 100%*** |
|  | GSYVQQGKGPKATVAKVPSD (112-131) | 11.17 *Cladosporium herbarum* | ***Penicillium* *manginii***100% |
|  | QIEEKFFGPPIPLYEPLDSG (399-418) | 11.5 *Fusarium proliferatum* | ***Pleopsidium* *flavum 94.7%*** |
|  | SHFETTTDDVRDLYKRATGE (433-452) | No allergen | ***Penicillium* *salami 95%*** |
| **ATP synthase F1, beta subunit** | PARDTGAPIKIPVGPGTLGR (113-132) | 11.9 *Rhodotorula mucilaginosa* | ***Pseudovirgaria* *hyperparasitica94.7%*** |
|  | ERITTTKKGSITSVQAVYVP (332-351) | 10.6 *Penicillium chrysogenum* | ***Tremellales* sp. 100%** |
|  | LAPYARGGKIGLFGGAGVGK (182-201) | 10.7 *Malassezia sympodialis* | ***Pneumocystis oryctolagi***100% |
|  | KVALVFGQMNEPPGARARVA (252-271) | 11.8 *Penicillium chrysogenum* | ***Pichia* *kudriavzevii 100%*** |

**Table S3:** T-cell epitope of proteins with ting property distance values with fungal allergen.

| **Protein name** | **T cell epitope** | **PD (fungal allergen)** | **Blastp results** |
| --- | --- | --- | --- |
| Proteasome component Pre9 | MTADANILI | *Cladosprium herbarum 6.79* | *Bifiguratus adelaidae 100%* |
|  | SAEKLYTLN | *Penicillium crustosum 6.58* | *auerobasidium subglaciale 100%* |
|  | EKLYTLNDN | *Cladosprium herbarum 7.05* | *auerobasidium subglaciale 100%* |
|  | HAGTALGIL | *Malassezia sympodialis 6.71* | *fulvia fulva 100%* |
| **Pyridoxine biosynthesis protein** | **AIVQAVTHY** | **No fungal allergen** | ***Bifiguratus adelaidae 100%*** |
|  | GCDGVFVGS | *Coprinus comatus 6.60* | *bifiguratus adelaidae 100%* |
|  | GRLPVVNFA | *Rhodotorula mucilaginosa 7.61* | *bifiguratus adelaidae 100%* |
|  | DPEIELRAY | *Cladosprium herbarum 7.93* | *Talaromyces marneffei 100%* |
| Spermidine synthase | GQIGFMVCC | *Penicillium chrysogenum (formerly P. notatum)8.58* | *verticillium longisporum 100%* |
|  | DYGTVLVLD | *Penicillium citrinum 7.26* | *chaetothyriales sp. CBS 134920 100%* |
|  | AMNLKVNQI | *Alternaria alternata 4.15* | *Monascus purpureus 100%* |
| Fructose-bisphosphate aldolase, class II | QVALEDFNT | *Cladosprium herbarum 6.28* | *Penicillium brasilianum 100%* |
|  | YGIPVVLHT | *Alternaria alternata 6.97* | *beauveria bassiana 100%* |
|  | PISPYFSIA | *Rhizopus oryzae 8.17* | *beauveria bassiana 100%* |
| Transaldolase | EVDARFSFD | *Cladosporium cladosporioides 0.00* | ***Verticillium longisporum****100%* |
|  | TTNPSLILA | *Fusarium proliferatum 0.00* | *Penicillium maclennan 100%* |
|  | KRDFSPEED | *Penicillium chrysogenum 3.29* | *Dothideomycetes Sp. JES 119 100%* |
| Adenosyl homocysteinase | AEGRLVNLG | *Penicillium chrysogenum 8.09* | *Kappamyces sp. JEL 0680 100%* |
|  | APAQKFKVA | *Alternaria alternata 5.88* | *Penicillium nordicum 100%* |
|  | ALGAEVTWT | *Malassezia furfur 6.98* | *xylaria flabelliformis 100%* |
|  | **AVQGYQVVT** | ***Fusarium culmorum 7.06*** | ***Dothideomycetes Sp. JES 119 100%*** |
| **Glucose-6 phosphate isomerase** | **DFSKNFLTE** | **No fungal allergen** | ***chlorociboria aeruginascens 100%*** |
|  | TLHFVSNID | *Coprinus comatus 6.62* | *chaetothyriales sp. cbs 135597 100%* |
|  | LFDFSKNFL | *Penicillium chrysogenum 6.06* | *chaetothyriales sp. cbs 135597 100%* |
|  | GLSVALYIG | *Alternaria alternata 7.57* | *Seirophora villosa 100%* |
| **ATP citrate lyase subunit (Acl), putatibe** | **GKEIKVETV** | **No fungal allergen** | ***Colletotrichum graminicola M1.001 88.89%*** |
|  | **YYININSVR** | **No fungal allergen** | ***Clonostachys rhizophaga 100%*** |
|  | AGGASVVY | *Penicillium chrysogenum 5.75* | *Penicillium chermesinum 100%* |
|  | ARAGKEIKV | *Malassezia sympodialis 6.87* | *Xylaria nigripes 100%* |
| 6 phosphogluconate dehydrogenase Gnd1 | DLGMPVTLI | *Malassezia furfur 7.47* | *Erysiphe pulchra 100%* |
|  | GKDIHVNWT | *Coprinus comatus 7.98* | *Fusarium solani 100%* |
|  | GGDIHVNWT | *Cladosprium herbarum 7.85* | *Dentiscutata erthropus 100%* |
|  | ALDLGMPVT | *Malassezia sympodialis 7.01* | *Ambrosiozyma monospora 100%* |
| Secretory pathway gdp dissociation inhibitor | GSDSLQLII | *Schizophyllum commune 6.58* | *Penicillium rolfsii 100%* |
|  | ARAGKEIKV | *Malassezia sympodialis 6.87* | *Xylaria nigripes 100%* |
|  | GTSAEVHFL | *Penicillium chrysogenum 6.37* | *chlorociboria aeruginascens 100%* |
|  | ASAGFVSEL | *Ulocladium chartarum (Alternaria chartarum) 6.07* | *Penicillium brasilianum 100%* |
| ATP synthase F1, beta subunit | KKDLTTNAR | *Penicillium citrinum 0.00* | ***Funneliformis geosporus 100%*** |
|  | DTERLIGDA | *Cladosprium herbarum 1.68* | *Clathrus columnatus 100%* |
|  | KKDLTTNAR | *Penicillium citrinum 0.00* | *Funneliformis geosporum 100%* |
|  | DTERLIGDA | *Cladosprium herbarum 1.68* | *Clathrus columnatus 100%* |

**Table S4:** Detailed data of Q-TOF MS/MS analysis of identified IgE reactive proteins of *Aspergillus fumigatus*.

| **Spot no.** | **Accession no.** | **Mass^1^ (kDa)** | **Mascot Score^2^** | **Peptide matched^3^** | **pI^4^** | **Protein name** | **MS/MS Analysis** |
| --- | --- | --- | --- | --- | --- | --- | --- |
| **1** | **XP_755878** | **28202** | **168** | **5** | **5.96** | **Sorbitol/xylulose reductase**  **Sou1-like** | **Sequence Coverage: 16%**  **1 MPIPVPAASS LLDLLSLKGK TVVVTGASGP RGMGIEAARG CAEMGANIAL**  **51 TYASRPEGGE KNAAEIAKTY GVKAKAYKCN VGDWESVQKL VADVIAEFGQ**  **101 IDAFIANAGR TASAGILDGS VNDWAEVIQT DLTGTFYCAK AVGPHFKERG**  **151 KGSFVITSSM SGHIANYPQE QTSYNVAKAG CIHFAKSLAN EWRDFARVNS**  **201 ISPGYIDTGL SDFVDKKTQD LWLSMIPMGR NGDAKELKGA YVYLCSDASS**  **251 YMTGTDLLID GGYCIR**  **2 - 18 867.5166 1733.0186 1733.0338 -0.0152 0 M.PIPVPAASSLLDLLSLK.G**  **2 - 20 640.4018 1918.1836 1918.1503 0.0333 1 M.PIPVPAASSLLDLLSLKGK.T**  **21 - 31 522.2896 1042.5646 1042.5771 -0.0125 0 K.TVVVTGASGPR.G**  **179 - 186 452.2237 902.4328 903.4273 -0.9944 0 K.AGCIHFAK.S Carboxymethyl (C)**  **187 - 193 438.2244 874.4342 874.4297 0.0046 0 K.SLANEWR.D (M)** |
| **2** | **XP_750785** | **28188** | **174** | **5** | **5.80** | **Proteasome component Pre9** | **Sequence Coverage: 16%**  **1 MSRRYDSRTT IFSPEGRLYQ VEYALEAISH AGTALGILAK DGIVLAAEKK**  **51 VTSKLLEQDT SAEKLYTLND NMICAVAGMT ADANILINYA RQAAQRYLLT**  **101 YGEEIPCEQL VRRLCDLKQG YTQHGGLRPF GVSFIYAGYD PLREFQLYQS**  **151 NPSGNYGGWK ATSVGANNAS AQSLLKQDYK EDCDLKEACA MAVKVLSKTM**  **201 DSTKLSSEKI EFATVGKTKE GKIYHHLWNA DEINALLKEH GLAKVDDEPE**  **251 AGDIK**  **9 - 17 504.2820 1006.5494 1006.5084 0.0411 0 R.TTIFSPEGR.L**  **9 - 17 504.2970 1006.5794 1006.5084 0.0711 0 R.TTIFSPEGR.L**  **55 - 64 567.3104 1132.6062 1132.5612 0.0451 0 K.LLEQDTSAEK.L**  **205 - 217 470.2768 1407.8086 1407.7609 0.0476 1 K.LSSEKIEFATVGK.T**  **245 - 255 594.2983 1186.5820 1186.5354 0.0467 0 K.VDDEPEAGDIK.-** |
| **3** | **XP_753827** | **32716** | **171** | **3** | **6.04** | **Pyridoxine biosynthesis protein** | **Sequence Coverage: 15%**  **1 MASNGTNGAS ASNSFTVKAG LAQMLKGGVI MDVVNAEQAR IAEEAGAAAV**  **51 MALERVPADI RAQGGVARMS DPSMIKEIMA AVTIPVMAKA RIGHFVECQI**  **101 LEAIGVDYID ESEVLTPADD VYHVKKHDYK VPFVCGCRNL GEALRRIAEG**  **151 AAMIRTKGEA GTGDVVEAVK HMRTVNSQIA RARSILQNST DPEIELRAYA**  **201 RELEVPYELL RETAEKGRLP VVNFAAGGVA TPADAALMMQ LGCDGVFVGS**  **251 GIFKSGDAKK RAKAIVQAVT HYKDPKVLAE VSEGLGEAMV GINVSQMPEA**  **301 DRLAKRGW**  **156 - 170 487.6082 1459.8028 1459.7518 0.0509 1 R.TKGEAGTGDVVEAVK.H**  **174 - 181 444.7613 887.5080 887.4825 0.0256 0 R.TVNSQIAR.A)**  **184 - 197 807.9560 1613.8974 1613.8260 0.0714 0 R.SILQNSTDPEIELR.A** |
| **4** | **XP_752719** | **33424** | **194** | **6** | **5.33** | **Spermidine synthase** | **Sequence Coverage: 17%**  **1 MSEITHPTIK DGWFSEQSDM WPGQAMNLKV NQILHHEKSK YQDVLVFEST**  **51 DYGTVLVLDN VIQCTERDEF SYQEMITHLA MNSHPNPKKV LVIGGGDGGV**  **101 LREVVKHESV EEAILCDIDE AVIRVSKKYL PGMSIGFQHP NVKVHIGDGF**  **151 QFLKERKNEF DVIITDSSDP EGPAESLFQK PYFELLRDAL RDGGVITTQG**  **201 SENQWLHLSL ITDLKKACKE VFPVAEYAYT TIPTYPSGQI GFMVCCKDAT**  **251 RNVREPVRTW SREEEERLCR YYNQDIHRAS FVLPNFARKA LDV**  **30 - 38 559.3132 1116.6118 1116.6040 0.0079 0 K.VNQILHHEK.S**  **89 - 102 447.2718 1338.7936 1338.7984 -0.0048 1 K.KVLVIGGGDGGVLR.E**  **90 - 102 606.3647 1210.7148 1210.7034 0.0114 0 K.VLVIGGGDGGVLR.E**  **144 - 154 630.8458 1259.6770 1259.6663 0.0108 0 K.VHIGDGFQFLK.E**  **271 - 278 554.7648 1107.5150 1107.5097 0.0053 0 R.YYNQDIHR.A**  **279 - 288 561.3143 1120.6140 1120.6029 0.0111 0 R.ASFVLPNFAR.K** |
| **5** | **XP_754776** | **43596** | **89** | **4** | **7.63** | **Inorganic diphosphatase** | **Sequence Coverage: 11%**  **1 MSPVALRMSS KALLPLSPLL RLSTASPALA RPCSPSPRTS ATLAPPTRQA**  **51 PPPSSLSSSS FSPSFSPQQA NLSSPSSSRV RSSVSSASTR AALLSRHFSS**  **101 YTPPQSPNMS YTVRKIGQAN TLEHRVYIEK DGVPISPFHD IPLYANPEQT**  **151 ILNMVVEIPR WTNAKQEISK EEFLNPIKQD VKKGKLRFVR NCFPHKGYLW**  **201 NYGAFPQTWE DPNVVHPETK AKGDNDPLDV CEIGELVGYP GQVKQVKVLG**  **251 VMALLDEEET DWKVIVIDIN DPLAPKLNDI EDVERHLPGL LRATNEWFRI**  **301 YKIPDGKPEN QFAFSGECKN KKYALDVIRE CADAWEKLIT GKSPRGDISL**  **351 ANTSVENSSD RADPAQLASI PKGENLPPAP IDGSIDKWFF ISGAAV**  **115 - 125 422.8985 1265.6737 1265.6840 -0.0103 1 R.KIGQANTLEHR.V**  **277 - 285 551.7785 1101.5424 1101.5302 0.0123 0 K.LNDIEDVER.H**  **346 - 361 832.9121 1663.8096 1663.7649 0.0447 0 R.GDISLANTSVENSSDR.A**  **362 - 372 555.8190 1109.6234 1109.6080 0.0154 0 R.ADPAQLASIPK.G** |
| **6** | **XP_754452** | **39766** | **144** | **8** | **5.55** | **Fructose-bisphosphate aldolase, class II** | **Sequence Coverage: 14%**  **1 MGILDKLSRK SGVIVGDDVL RLFEYAQEKN FAIPAVNVTS SSTVVACLEA**  **51 ARDQNCPIIL QVSQGGAAYF AGKGVSNDGQ KASIAGSIAA AHYIRSIAPS**  **101 YGIPVVLHTD HCAKKLLPWL DGMLDEDERY FKQHGEPLFS SHMIDLSEEP**  **151 VDYNIETTAK YLKRAAPMKQ WLEMEIGITG GEEDGVNNED VDNNSLYTQP**  **201 EDILAIYNAL APISPYFSIA AGFGNVHGVY KPGNVRLHPE LLSKHQAYVK**  **251 EKIGSNKDKP VYFVFHGGSG STKEEYKQAI SYGVVKVNLD TDMQYAYMSG**  **301 VRDYILNKKD YLMSAVGNPD GEDKPNKKYF DPRVWVREGE KTMSKRVQVA**  **351 LEDFNTAGQL**  **10 - 21 419.9008 1256.6806 1256.7089 -0.0283 1 R.KSGVIVGDDVLR.L**  **10 - 21 419.9025 1256.6857 1256.7089 -0.0232 1 R.KSGVIVGDDVLR.L**  **10 - 21 629.3571 1256.6996 1256.7089 -0.0092 1 R.KSGVIVGDDVLR.L**  **11 - 21 565.3026 1128.5906 1128.6139 -0.0233 0 K.SGVIVGDDVLR.L**  **22 - 29 514.2482 1026.4818 1026.5022 -0.0203 0 R.LFEYAQEK.N**  **278 - 286 482.7595 963.5044 963.5389 -0.0345 0 K.QAISYGVVK.V**  **303 - 309 447.2494 892.4842 892.5018 -0.0175 1 R.DYILNKK.D**  **346 - 360 830.9331 1659.8516 1659.8580 -0.0064 1 K.RVQVALEDFNTAGQL.-** |
| **7** | **XP_753716** | **35426** | **402** | **16** | **6.04** | **Transaldolase** | **Sequence Coverage: 37%**  **1 MSSALEQLKA TGTVVVCDSG DFATIGKYKP QDATTNPSLI LAASKKPEYA**  **51 SLIDAAVQKG KKEGKTLDEQ VDATLDNLLV EFGKKILEII PGKVSTEVDA**  **101 RFSFDTQASV DKALHIVKLY EQQGISKDRI LIKIASTWEG IKAAHILQSQ**  **151 HGINCNLTLM FSLVQAIAAA EAGAFLISPF VGRILDWYKA AHKRDFSPEE**  **201 DPGVKSVQSI FNYYKKHGYK TIVMGASFRN TGEITELAGC DYLTISPNLL**  **251 EELYNSTASV PKKLDAASAA SLDIPKRSYI NDEAAFRFDF NEEAMAVEKL**  **301 REGISKFAAD AVTLKELLKQ KIQA**  **28 - 45 640.0280 1917.0622 1917.0207 0.0415 0 K.YKPQDATTNPSLILAASK.K**  **28 - 45 959.5454 1917.0762 1917.0207 0.0555 0 K.YKPQDATTNPSLILAASK.K**  **46 - 59 511.6237 1531.8493 1531.8246 0.0247 0 K.KPEYASLIDAAVQK.G**  **86 - 101 580.6792 1739.0158 1738.9829 0.0329 1 K.ILEIIPGKVSTEVDAR.F**  **94 - 101 438.7287 875.4428 875.4349 0.0080 0 K.VSTEVDAR.F**  **102 - 112 622.8083 1243.6020 1243.5721 0.0300 0 R.FSFDTQASVDK.A**  **119 - 127 533.2903 1064.5660 1064.5502 0.0158 0 K.LYEQQGISK.D**  **119 - 129 446.2392 1335.6958 1335.6783 0.0175 1 K.LYEQQGISKDR.I**  **134 - 142 502.7865 1003.5584 1003.5338 0.0246 0 K.IASTWEGIK.A**  **195 - 205 610.2894 1218.5642 1218.5405 0.0238 0 R.DFSPEEDPGVK.S**  **206 - 215 624.8318 1247.6490 1247.6186 0.0304 0 K.SVQSIFNYYK.K**  **206 - 216 459.5841 1375.7305 1375.7136 0.0169 1 K.SVQSIFNYYKK.H**  **206 - 216 688.8820 1375.7494 1375.7136 0.0359 1 K.SVQSIFNYYKK.H**  **278 - 287 593.2908 1184.5670 1184.5462 0.0209 0 R.SYINDEAAFR.F**  **307 - 315 468.2704 934.5262 934.5124 0.0139 0 K.FAADAVTLK.E**  **307 - 319 473.6196 1417.8370 1417.8180 0.0189 1 K.FAADAVTLKELLK.Q** |
| **8** | **Q96X30** | **47276** | **772** | **19** | **5.39** | **Enolase/ Asp F22** | **Sequence Coverage: 40%**  **1 MPISKIHARS VYDSRGNPTV EVDVVTETGL HRAIVPSGAS TGQHEAHELR**  **51DGDKTQWGGK GVLKAVKNVN ETIGPALIKE NIDVKDQSKV DEFLNKLDGT**  **101 ANKSNLGANA ILGVSLAVAK AGAAEKGVPL YAHISDLAGT KKPYVLPVPF**  **151 QNVLNGGSHA GGRLAFQEFM IVPDSAPSFS EALRQGAEVY QKLKALAKKK**  **201 YGQSAGNVGD EGGVAPDIQT AEEALDLITE AIEQAGYTGK IKIAMDVASS**  **251 EFYKADVKKY DLDFKNPESD PSKWLTYEQL ADLYKSLAAK YPIVSIEDPF**  **301 AEDDWEAWSY FYKTSDFQIV GDDLTVTNPG RIKKAIELKS CNALLLKVNQ**  **351 IGTLTESIQA AKDSYADNWG VMVSHRSGET EDVTIADIAV GLRSGQIKTG**  **401 APCRSERLAK LNQILRIEEE LGENAVYAGS KFRTAVNL**  **16 - 32 608.3074 1821.9004 1821.9221 -0.0217 0 R.GNPTVEVDVVTETGLHR.A**  **16 - 32 608.3075 1821.9007 1821.9221 -0.0214 0 R.GNPTVEVDVVTETGLHR.A**  **33 - 50 620.6436 1858.9090 1858.9286 -0.0196 0 R.AIVPSGASTGQHEAHELR.D**  **68 - 79 634.8556 1267.6966 1267.7136 -0.0169 0 K.NVNETIGPALIK.E**  **80 - 89 588.2962 1174.5778 1174.5830 -0.0051 1 K.ENIDVKDQSK.V**  **90 - 103 521.9288 1562.7646 1562.7940 -0.0295 1 K.VDEFLNKLDGTANK.S**  **90 - 103 782.4031 1562.7916 1562.7940 -0.0024 1 K.VDEFLNKLDGTANK.S**  **104 - 120 799.4644 1596.9142 1596.9199 -0.0056 0 K.SNLGANAILGVSLAVAK.A**  **104 - 120 799.4797 1596.9448 1596.9199 0.0250 0 K.SNLGANAILGVSLAVAK.A**  **127 - 141 514.6090 1540.8052 1540.8249 -0.0198 0 K.GVPLYAHISDLAGTK.K**  **127 - 141 514.6102 1540.8088 1540.8249 -0.0162 0 K.GVPLYAHISDLAGTK.K**  **185 - 192 461.7259 921.4372 921.4556 -0.0183 0 R.QGAEVYQK.L**  **314 - 331 967.9717 1933.9288 1933.9382 -0.0093 0 K.TSDFQIVGDDLTVTNPGR.I**  **314 - 331 967.9889 1933.9632 1933.9382 0.0251 0 K.TSDFQIVGDDLTVTNPGR.I**  **348 - 362 524.9501 1571.8285 1571.8519 -0.0234 0 K.VNQIGTLTESIQAAK.D**  **348 - 362 786.9335 1571.8524 1571.8519 0.0006 0 K.VNQIGTLTESIQAAK.D**  **377 - 393 873.4526 1744.8906 1744.8843 0.0063 0 R.SGETEDVTIADIAVGLR.S**  **417 - 431 804.8896 1607.7646 1607.7678 -0.0032 0 R.IEEELGENAVYAGSK.F**  **417 - 431 804.8900 1607.7654 1607.7678 -0.0024 0 R.IEEELGENAVYAGSK.F** |
| **9** | **XP_752379** | **48459** | **220** | **6** | **5.82** | **Adenosylhomocysteinase** | **Sequence Coverage: 12%**  **1 MAAPAQKFKV ADISLAAFGR REIELAEIEM PGLMAIRRKY AEDQPLKGAR**  **51 IAGCLHMTIQ TAVLIETLTA LGAEVTWTSC NIFSTQDHAA AAIAASGVPV**  **101 FAWKGETEEE YQWCLEQQLN AFKDGQKLNL ILDDGGDLTA LVHSKYPEML**  **151 KGCYGVSEET TTGVHHLYRM LKEGKLLVPA INVNDSVTKS KFDNLYGCRE**  **201 SLIDGIKRAT DVMIAGKVAV VAGYGDVGKG CADALRSMGA RVLVTEIDPI**  **251 NALQAAVQGY QVVTMEEAAP QGQIFVTTTG CRDILVGKHF EVMRNDAIVC**  **301 NIGHFDIEID VAWLKANAKS VQNIKPQVDR YLMPNGRHII LLAEGRLVNL**  **351 GCATGHSSFV MSCSFSNQVL AQIALFKAED EAFGKKYVEF GTTGKKPVGV**  **401 YVLPKILDEQ VALLHLEHVN AKLSKLTPVQ AEYLGLDIAG PFKSDM**  **10 - 20 560.3098 1118.6050 1118.6084 -0.0034 0 K.VADISLAAFGR.R**  **176 - 189 741.9341 1481.8536 1481.8453 0.0083 0 K.LLVPAINVNDSVTK.S**  **320 - 330 428.5662 1282.6768 1282.6993 -0.0226 0 K.SVQNIKPQVDR.Y**  **320 - 330 642.3569 1282.6992 1282.6993 -0.0001 0 K.SVQNIKPQVDR.Y**  **338 - 346 511.3066 1020.5986 1020.6080 -0.0093 0 R.HIILLAEGR.L**  **387 - 395 501.2462 1000.4778 1000.4866 -0.0087 0 K.YVEFGTTGK.K** |
| **10** | **EDP54506** | **61335** | **213** | **6** | **5.85** | **Glucose-6-phosphate isomerase** | **Sequence Coverage: 14%**  **1 MPGFSQATEL GAWKELQEHH NSLGRNIVLK EYFEKDPQRF EKFSRTFANP**  **51 VDNTEILFDF SKNFLTEETL ALLVKLAREA GVEELRDAMF KGDPINFTED**  **101 RAVYHVALRN VTNEPMQVNG KSVVEDVNSV LEHMKEFTEQ VRSGEWKGYT**  **151 GKKITTIINI GIGGSDLGPV MVTEALKPYG AEDMTLHFVS NIDGSHIAEA**  **201 LKHSDPETTL FLIASKTFTT AETTTNANSA KKWFLESAKD EAHIAKHFVA**  **251 LSTNEEEVTK FGIDKKNMFG FASWVGGRYS VWSAIGLSVA LYIGFDNFHQ**  **301 FLAGAHAMDK HFRETPLEQN IPVLGGLLSV WYSDFFGAQT HLVAPFDQYL**  **351 HRFPAYLQQL SMESNGKAIT RTGEYVKYTT GPILFGEPAT NAQHSFFQLL**  **401 HQGTKLIPSD FIMAAESHNP VEGGKHQRML ASNFLAQSEA LMVGKTPEQV**  **451 KTEGAPDNLV PHKTFLGNRP TTSILAQKIT PSTLGALIAY YEHLTFTEGA**  **501 VWNINSFDQW GVELGKVLAK KIQKELETPG AGGDHDASTS GLLLAFKKKA**  **551 NLA**  **46 - 62 979.4716 1956.9286 1956.9469 -0.0183 0 R.TFANPVDNTEILFDFSK.N**  **92 - 101 582.2633 1162.5120 1162.5255 -0.0134 0 K.GDPINFTEDR.A**  **110 - 121 673.8157 1345.6168 1345.6296 -0.0128 0 R.NVTNEPMQVNGK.S Oxidation (M)**  **203 - 216 779.9022 1557.7898 1557.8039 -0.0140 0 K.HSDPETTLFLIASK.T**  **217 - 231 779.3685 1556.7224 1556.7318 -0.0094 0 K.TFTTAETTTNANSAK.K**  **247 - 260 535.2649 1602.7729 1602.7889 -0.0161 0 K.HFVALSTNEEEVTK.F** |
| **11** | **XP_750954** | **52885** | **471** | **16** | **5.88** | **ATP citrate lyase subunit**  **(Acl), putatibe** | **Sequence Coverage: 29%**  **1 MSAKSILEAD GKAILNYHLT RAPVIKPTPL PPSSTHNPPP RLASIYFPED**  **51 AAVKDVLDQT EVVYPWLLTP GAKFVAKPDQ LIKRRGKSGL LALNKTWPEA**  **101 REWIEARAGK EIKVETVTGV LRQFLVEPFV PHPQETEYYI NINSVREGDW**  **151 ILFTHEGGVD VGDVDAKAEK LLIPVNLRNY PSNEEIAAAL LSKVPKGVHN**  **201 VLVDFISRLY AVYVDCQFTY LEINPLVVIP NAEGTSAEVH FLDLAAKLDQ**  **251 TAEFECGTKW AIARSPANLG LATVPQTDGK VNIDAGPPME FPAPFGRELS**  **301 KEEKFISDMD AKTGASLKLT VLNANGRIWT LVAGGGASVV YADAIASAGF**  **351 VSELANYGEY SGAPTETQTY NYAKTVLDLM LRAPMHPDGK VLFIGGGIAN**  **401 FTNVASTFKG VIRAIREVAP VLNEHNVQIW VRRAGPNYQE GLKNIKAVGE**  **451 ELGLKMHVYG PDMHVSGIVP LALLGKKTDI KEFGSA**  **13 - 21 550.8359 1099.6572 1099.6138 0.0434 0 K.AILNYHLTR.A**  **74 - 83 579.8720 1157.7294 1157.6808 0.0486 0 K.FVAKPDQLIK.R**  **111 - 122 672.4186 1342.8226 1342.7820 0.0406 1 K.EIKVETVTGVLR.Q**  **171 - 178 469.3264 936.6382 936.6120 0.0262 0 K.LLIPVNLR.N**  **171 - 178 469.3290 936.6434 936.6120 0.0314 0 K.LLIPVNLR.N**  **179 - 193 810.4426 1618.8706 1618.8202 0.0505 0 R.NYPSNEEIAAALLSK.V**  **179 - 193 810.4478 1618.8810 1618.8202 0.0609 0 R.NYPSNEEIAAALLSK.V**  **197 - 208 452.6009 1354.7809 1354.7357 0.0451 0 K.GVHNVLVDFISR.L**  **197 - 208 678.4030 1354.7914 1354.7357 0.0557 0 K.GVHNVLVDFISR.L**  **265 - 280 784.9504 1567.8862 1567.8206 0.0657 0 R.SPANLGLATVPQTDGK.V**  **265 - 280 784.9531 1567.8916 1567.8206 0.0711 0 R.SPANLGLATVPQTDGK.V**  **281 - 297 915.9880 1829.9614 1829.8771 0.0844 0 K.VNIDAGPPMEFPAPFGR.E Oxidation (M)**  **305 - 312 471.7370 941.4594 941.4164 0.0430 0 K.FISDMDAK.T Oxidation (M)**  **319 - 327 479.2922 956.5698 956.5403 0.0295 0 K.LTVLNANGR.I**  **375 - 382 488.7997 975.5848 975.5423 0.0426 0 K.TVLDLMLR.A Oxidation (M)**  **391 - 409 978.5823 1955.1500 1955.0517 0.0984 0 K.VLFIGGGIANFTNVASTFK.G** |
| **12** | **XP_750696** | **55764** | **830** | **16** | **5.86** | **6-phosphogluconate dehydrogenase Gnd1** | **Sequence Coverage: 31%**  **1 MSTQAVARLA GINVGAPARP LPSADFGLIG LAVMGQNLIL NVADHGFTVC**  **51 AYNRTTSKVD RFLANEAKGK SIVGAHSVEE FCAKLKRPRR IMLLVMAGKP**  **101 VDDFIESLLP HLEEGDIIID GGNSHFPDSN RRTKYLKEKG IRFVGSGVSG**  **151 GEEGARYGPS LMPGGNEEAW PFIKDIFQSI AAKSDGEACC DWVGDEGAGH**  **201 FVKMVHNGIE YGDMQLICEA YDIMKRGLGM PVNEIADVFA KWNKGVLDSF**  **251 LIEITRDVLY FNDNDGTPLV EKILDKAGQK GTGKWTAINA LDLGMPVTLI**  **301 GEAVFARCLS AIKDERIRAS SLLDGPTPQF TGDKQAFIDD LEQALYASKI**  **351 ISYAQGFMLI QEAAREYGWK LNKPSIALMW RGGCIIRSVF LKDITNAYRN**  **401 NPDLENLLFD DFFKAAIQKA QQGWRNVVSK GALWGIPTPA FSTALSFYDG**  **451 YRTRDLPANL LQAQRDYFGA HTFRVKPECA NENYPEGKDI HVNWTGRGGD**  **501 VSASTYVV**  **143 - 156 654.8308 1307.6470 1307.6106 0.0364 0 R.FVGSGVSGGEEGAR.Y**  **175 - 183 496.7874 991.5602 991.5338 0.0264 0 K.DIFQSIAAK.S**  **227 - 241 788.9334 1575.8522 1575.7967 0.0556 0 R.GLGMPVNEIADVFAK.W Oxidation (M)**  **245 - 256 681.9071 1361.7996 1361.7555 0.0442 0 K.GVLDSFLIEITR.D**  **257 - 272 919.9794 1837.9442 1837.8734 0.0708 0 R.DVLYFNDNDGTPLVEK.I**  **319 - 334 817.4396 1632.8646 1632.7995 0.0651 0 R.ASSLLDGPTPQFTGDK.Q**  **335 - 349 856.4639 1710.9132 1710.8464 0.0668 0 K.QAFIDDLEQALYASK.I**  **350 - 365 609.6726 1825.9960 1825.9396 0.0564 0 K.IISYAQGFMLIQEAAR.E Oxidation (M)**  **350 - 365 914.0105 1826.0064 1825.9396 0.0668 0 K.IISYAQGFMLIQEAAR.E Oxidation (M)**  **388 - 399 476.2736 1425.7990 1425.7616 0.0374 1 R.SVFLKDITNAYR.N**  **393 - 399 426.7232 851.4318 851.4137 0.0181 0 K.DITNAYR.N**  **400 - 414 920.9688 1839.9230 1839.8679 0.0551 0 R.NNPDLENLLFDDFFK.A**  **453 - 465 499.2953 1494.8641 1494.8266 0.0374 1 R.TRDLPANLLQAQR.D**  **455 - 465 619.8625 1237.7104 1237.6779 0.0326 0 R.DLPANLLQAQR.D**  **455 - 465 619.8661 1237.7176 1237.6779 0.0398 0 R.DLPANLLQAQR.D**  **466 - 474 557.2783 1112.5420 1112.5040 0.0381 0 R.DYFGAHTFR.V** |
| **13** | **XP_755448** | **52247** | **124** | **3** | **5.33** | **Secretory pathway gdp dissociation inhibitor** | **Sequence Coverage: 7%**  **1 MEEIAPEYDV VVLGTGLTEC VLSGVLSVKG NKVLHIDRND HYGGEAASVN**  **51 IETLFKKYGN VRPGEEPWKK YGRVNDWNID LVPKLLMANG ELTNILVSTD**  **101 VTRYLEFKQI AGSYVQQGKG PKATVAKVPS DAGEALRSSL MGMFEKRRAK**  **151 KFLEWVGEFK EDDPATHQGL NVAQCTMKEV YDKFGLEDNT RDFVGHSMAL**  **201 YPSDDYITTP GMAVETIHRI RLYVNSMARY GKSPYIYPLY GLGELPQGFA**  **251 RLSAIYGGTY MLNTSVDEVL YDESGKVSGI KATMKDRDDN SEAMKFTTKT**  **301 KKIIADPSYF PGKVRVTGYL LKAICILNHP IEKTDGSDSL QLIIPQSQVG**  **351 RKHDVYIAMV SSAHNVCPKG YYIAIVSTIA ETDANHHLEL EPGFERLGQI**  **401 EEKFFGPPIP LYEPLDSGEK DNIFISKSYD ATSHFETTTD DVRDLYKRAT**  **451 GEELVVEGLR EDQRLAED**  **128 - 137 507.7652 1013.5158 1013.5142 0.0017 0 K.VPSDAGEALR.S**  **303 - 313 604.3301 1206.6456 1206.6285 0.0172 0 K.IIADPSYFPGK.V**  **449 - 460 636.8434 1271.6722 1271.6721 0.0001 0 R.ATGEELVVEGLR.E** |
| **14** | **XP_752631** | **79995** | **351** | **9** | **5.08** | **Hsp70 chaperone Hsp88** | **Sequence Coverage: 12%**  **1 MSVVGIDFGA QSTKVGVARN KGIDIITNEV SNRSTPTLVG FGTRSRHIGE**  **51 GAKTQEMSNL KNTVGNLKRL IGRSFNDPDV EIEQKYTSAA ICDVNGQAGV**  **101 EVSYLGKKEK FSATQLAAMY LTKIRDITSK ELKLPVSDVT ISVPAWFTDA**  **151 QRRAMIDAGE IAGLKVLRLI NDTTATALGY GITKLDLPGP EEKPRRVMFV**  **201 DIGHSDYTAS VVEFRKGELN VKATAYDRHF GGRDFDIALT EHFADEFKEK**  **251 FKIDVRSNPK AYARTVAAAE KMKKVLSANP AAPMSIESLM EDVDVRSIVK**  **301 REELETMVKP LLERVTIPIE EALAEAKLKP EDIDTIEMVG GCTRVPAIKE**  **351 AISKFFGKTL SFTLNQDEAI ARGCAFSCAI LSPVFRVRDF SVHDIVNYPI**  **401 EFTWEQSADI PDEDTSLTVF NRGNVMPSTK ILTFYRKQPF DLEARYAKPD**  **451 MLPGKINPWI GRFSVKGVKA DANDDFMICK LKARLNLHGI LNVESGYYVE**  **501 DVEVEEPVDE DKMDTDAPEG EQPKKTRKVK KQVRKGDLPI STGTNSVDQT**  **551 VKETWIEREN AMYMEDKLIA ETDEKKNELE STIYELRDKI DGVYAEFASE**  **601 EEKDKLRAKL TDMEDWLYEE GEDTTKSVYV AKLDEIRFIA GPIVQRYKEK**  **651 IEAERQAILK AQEEEAAKKR AEEEAKRKAE EEAKKAEETK PDEEMKDAPA**  **701 EGEAAPAEGE EKQQ**  **20 - 33 524.9613 1571.8621 1571.8267 0.0354 1 R.NKGIDIITNEVSNR.S**  **22 - 33 665.8726 1329.7306 1329.6888 0.0418 0 K.GIDIITNEVSNR.S**  **34 - 44 568.3196 1134.6246 1134.6033 0.0213 0 R.STPTLVGFGTR.S**  **169 - 184 826.4747 1650.9348 1650.8828 0.0520 0 R.LINDTTATALGYGITK.L**  **185 - 195 417.5660 1249.6762 1249.6666 0.0095 0 K.LDLPGPEEKPR.R**  **315 - 327 692.4059 1382.7972 1382.7657 0.0316 0 R.VTIPIEEALAEAK.L**  **315 - 327 692.4122 1382.8098 1382.7657 0.0442 0 R.VTIPIEEALAEAK.L**  **359 - 372 789.9340 1577.8534 1577.8049 0.0485 0 K.TLSFTLNQDEAIAR.G** |
| **15** | **XP_747926** | **80590** | **580** | **11** | **4.95** | **Asp f12/Heat shock protein P90/Molecular chaperone and allergen Mod-E/Hsp90/Hsp1** | **Sequence Coverage: 20%**  **1 MSSETFEFQA EISQLLSLII NTVYSNKEIF LRELISNASD ALDKIRYQSL**  **51 SDPTKLDTGK DLRIDIIPDK ENKTLTIRDT GIGMTKADLI NNLGTIARSG**  **101 TKQFMEALSA GADISMIGQF GVGFYSAYLV ADRVTVVSKN NDDEQYIWES**  **151 AAGGTFTLTQ DTEGEQLGRG TKIILHLKDE QTDYLNESRI KEVVRKHSEF**  **201 ISYPIYLHVL KETEKEVPDE EAEETKEEED EEKKAKIEEV DDEEEEEKKK**  **251 KKKTKTVKES KIEEEELNKT KPIWTRNPAD ITQEEYASFY KSLSNDWEDH**  **301 LAVKHFSVEG QLEFRAILYV PKRAPFDLFE TKKTKNNIKL YVRRVFITDD**  **351 ATDLIPEWLG FIKGVVDSED LPLNLSRETL QQNKIMKVIK KNIVKKTLEL**  **401 FNEIAEDREQ FDKFYSAFSK NIKLGIHEDA QNRQTLAKLL RYQSTKSGDE**  **451 ATSLADYVTR MPEHQKQIYY ITGESIKAVA KSPFLDSLKQ KNFEVLFLVD**  **501 PIDEYAFTQL KEFDGKKLVD ITKDFELEET EEEKAEREKE EKEYENLAKS**  **551 LKNILGDKVE KVVVSHKLVG SPCAIRTGQF GWSANMERIM KAQALRDTSM**  **601 SSYMSSKKTF EISPKSSIIK ELKKKVEADG ENDRTVKSIT QLLFETSLLV**  **651 SGFTIEEPAS FAERIHKLVS LGLNIDEEAE TTEEKATEEA APAEATTGES**  **701 AMEEVD**  **33 - 44 638.3338 1274.6530 1274.6354 0.0177 0 R.ELISNASDALDK.I**  **87 - 98 635.8681 1269.7216 1269.7041 0.0176 0 K.ADLINNLGTIAR.S**  **173 - 189 696.3751 2086.1035 2086.0694 0.0340 1 K.IILHLKDEQTDYLNESR.I**  **277 - 291 888.4280 1774.8414 1774.8050 0.0365 0 R.NPADITQEEYASFYK.S**  **323 - 332 612.3378 1222.6610 1222.6346 0.0264 1 K.RAPFDLFETK.K**  **364 - 377 757.4090 1512.8034 1512.7784 0.0251 0 K.GVVDSEDLPLNLSR.E**  **397 - 408 725.3771 1448.7396 1448.7147 0.0250 0 K.TLELFNEIAEDR.E**  **424 - 433 576.8013 1151.5880 1151.5683 0.0197 0 K.LGIHEDAQNR.Q**  **447 - 460 742.8643 1483.7140 1483.6791 0.0350 0 K.SGDEATSLADYVTR.M**  **467 - 477 657.8640 1313.7134 1313.6867 0.0268 0 K.QIYYITGESIK.A**  **668 - 685 995.5217 1989.0288 1988.9790 0.0499 0 K.LVSLGLNIDEEAETTEEK.A** |
| **16** | **XP_750490** | **69618** | **693** | **21** | **5.09** | **Molecular chaperone Hsp70** | **Sequence Coverage: 26%**  **1 MAPAVGIDLG TTYSCVGVFR DDRIEIIAND QGNRTTPSFV AFTDTERLIG**  **51 DAAKNQVAMN PHNTVFDAKR LIGRRFQDAE VQSDMKHWPF KVVEKGGKPI**  **101 IEVEFKGETK QFTPEEISSM VLTKMRETAE AYLGGTVNNA VITVPAYFND**  **151 SQRQATKDAG LIAGLNVLRI INEPTAAAIA YGLDKKAEGE RNVLIFDLGG**  **201 GTFDVSLLTI EEGIFEVKAT AGDTHLGGED FDNRLVNHFV NEFKRKHKKD**  **251 LTTNARALRR LRTACERAKR TLSSAAQTSI EIDSLFEGID FYTSITRARF**  **301 EELCQDLFRS TMEPVERVLR DAKLDKSSVH EIVLVGGSTR IPKIQRLVAD**  **351 FFNKEANKSI NPDEAVAYGA AVQAAILSGD TSSKSTNEIL LLDVAPLSLG**  **401 IETAGGVMTP LIKRNTTIPT KKSETFSTYS DNQPGVLIQV YEGERARTKD**  **451 NNLLGKFELT GIPPAPRGVP QIEVTFDVDA NGIMNVSAVE KGTGKTNKIT**  **501 ITNDKGRLSK EEIERMLADA EKYKEEDEAE AARIQAKNGL ESYAYSLKNT**  **551 ISEGKLNISD ADKEKVSSKV EEIISWLDNN QTATKDEYES QQKELESVAN**  **601 PIISAAYGGA AGAAPGGAAP GGATRTADEV EERPEELD**  **24 - 34 621.8320 1241.6494 1241.6364 0.0131 0 R.IEIIANDQGNR.T**  **35 - 47 736.3662 1470.7178 1470.6991 0.0188 0 R.TTPSFVAFTDTER.L**  **158 - 169 606.3650 1210.7154 1210.7034 0.0121 0 K.DAGLIAGLNVLR.I**  **170 - 185 553.9749 1658.9029 1658.8879 0.0150 0 R.IINEPTAAAIAYGLDK.K**  **170 - 185 830.4614 1658.9082 1658.8879 0.0204 0 R.IINEPTAAAIAYGLDK.K**  **170 - 186 596.6722 1786.9948 1786.9828 0.0119 1 R.IINEPTAAAIAYGLDKK.A**  **219 - 234 559.2504 1674.7294 1674.7234 0.0060 0 K.ATAGDTHLGGEDFDNR.L**  **219 - 234 838.3850 1674.7554 1674.7234 0.0320 0 K.ATAGDTHLGGEDFDNR.L**  **235 - 244 623.8408 1245.6670 1245.6506 0.0164 0 R.LVNHFVNEFK.R**  **235 - 244 623.8414 1245.6682 1245.6506 0.0176 0 R.LVNHFVNEFK.R**  **235 - 244 416.2370 1245.6892 1245.6506 0.0386 0 R.LVNHFVNEFK.R**  **327 - 340 480.9323 1439.7751 1439.7733 0.0018 0 K.SSVHEIVLVGGSTR.I**  **327 - 340 720.9034 1439.7922 1439.7733 0.0190 0 K.SSVHEIVLVGGSTR.I**  **347 - 354 477.2532 952.4918 952.5018 -0.0100 0 R.LVADFFNK.E**  **347 - 354 477.2624 952.5102 952.5018 0.0084 0 R.LVADFFNK.E**  **359–384 845.7729 2534.2969 2534 .2500 0.0469 0 K.SINPDEAVAYGAAVQAAILSGDTSSK.S**  **457 - 467 599.3398 1196.6650 1196.6553 0.0097 0 K.FELTGIPPAPR.G**  **457 - 467 599.3604 1196.7062 1196.6553 0.0509 0 K.FELTGIPPAPR.G**  **508 - 515 502.2781 1002.5416 1002.5345 0.0071 1 R.LSKEEIER.M**  **538 - 548 622.8209 1243.6272 1243.6084 0.0188 0 K.NGLESYAYSLK.N)**  **626 - 638 766.3547 1530.6948 1530.6685 0.0263 0 R.TADEVEERPEELD.-** |
| **17** | **XP_753589** | **55586** | **382** | **13** | **5.30** | **ATP synthase F1, beta subunit** | **Sequence Coverage: 31%**  **1 MFKSGLARTF GRAAFARPSP VARRAIQPVR YNGLPSLARF ASSEAGSVGK**  **51 IHQVIGAVVD VKFDGDNLPA ILNAIETENN GQKLVLEVSQ HLGENVVRTI**  **101 AMDGTEGLTR GAPARDTGAP IKIPVGPGTL GRIVNVTGDP IDERGPIKAT**  **151 KFAPIHAEAP EFTEQSTSAE ILVTGIKVVD LLAPYARGGK IGLFGGAGVG**  **201 KTVFIQELIN NIAKAHGGYS VFTGVGERTR EGNDLYHEMQ ETGVIQLEGE**  **251 SKVALVFGQM NEPPGARARV ALTGLTIAEY FRDEEGQDVL LFIDNIFRFT**  **301 QAGSEVSALL GRIPSAVGYQ PTLAVDMGGM QERITTTKKG SITSVQAVYV**  **351 PADDLTDPAP ATTFAHLDAT TVLSRGISEL GIYPAVDPLD SKSRMLDPRI**  **401 VGEEHYAVAT RVQQMLQEYK SLQDIIAILG MDELSEADKL TVERARKLQR**  **451 FLSQPFTVAQ VFTGIEGKLV DLKDTIRSFK AIINGEGDDL PEAAFYMVGD**  **501 FESARAKGEK ILAELEGKA**  **84 - 98 564.6611 1690.9615 1690.9366 0.0249 0 K.LVLEVSQHLGENVVR.T**  **116 - 132 550.3268 1647.9586 1647.9308 0.0278 1 R.DTGAPIKIPVGPGTLGR.I**  **133 - 144 664.3539 1326.6932 1326.6779 0.0153 0 R.IVNVTGDPIDER.G**  **133 - 148 574.9902 1721.9488 1721.9312 0.0176 1 R.IVNVTGDPIDERGPIK.A**  **178 - 187 558.8283 1115.6420 1115.6339 0.0082 0 K.VVDLLAPYAR.G**  **191 - 201 488.2902 974.5658 974.5549 0.0109 0 K.IGLFGGAGVGK.T**  **202 - 214 751.9456 1501.8766 1501.8504 0.0262 0 K.TVFIQELINNIAK.A**  **215 - 228 479.5700 1435.6882 1435.6844 0.0037 0 K.AHGGYSVFTGVGER.T**  **299 - 312 718.3895 1434.7644 1434.7467 0.0178 0 R.FTQAGSEVSALLGR.I**  **376 - 392 887.4830 1772.9514 1772.9196 0.0318 0 R.GISELGIYPAVDPLDSK.S**  **400 - 411 448.9020 1343.6842 1343.6834 0.0008 0 R.IVGEEHYAVATR.V**  **451 - 468 985.0487 1968.0828 1968.0357 0.0472 0 R.FLSQPFTVAQVFTGIEGK.L**  **511 - 519 472.2781 942.5416 942.5386 0.0031 1 K.ILAELEGKA.-** |
| **18** | **XP_751171** | **85475** | **361** | **16** | **6.26** | **Mitochondrial aconitate hydratase** | **Sequence Coverage: 21%**  **1 MISTRLARAG ALAPKSRLFL GTRAFATVGD SPLDKKVEMA NTEKGNYINY**  **51 KKMSENLDIV RRRLQRPLTY AEKVLYSHLD DPHGQEIERG KSYLKLRPDR**  **101 VACQDATAQM AILQFMSAGM PSVATPTTVH CDHLIEAQVG GDKDLARANE**  **151 INKEVYDFLA SATAKYNIGF WKPGSGIIHQ IVLENYAFPG GLMIGTDSHT**  **201 PNAGGLAMAA IGVGGADAVD VMAGLPWELK APKVIGVKLT GEMSGWTTPK**  **251 DVILKVAGLL TVKGGTGAII EYHGPGVTSL SCTGMGTICN MGAEIGATTS**  **301 MFPFNDRMYD YLKATKRQHI GDFAREYAKE LREDEGAEYD QLIEINLSEL**  **351 EPHINGPFTP DLATPISKFK EAVETNKWPE ELKVGLIGSC TNSSYEDMSR**  **401 AASIARDALN HGLKAKSLFT VTPGSEQIRA TIERDGQLQT LEEFGGVILA**  **451 NACGPCIGQW DRRDVKKGEP NSIISSYNRN FTGRNDANPA THAFVASPDL**  **501 VVAMTIAGTL KFNPLTDKLK DKDGNEFLLQ PPTGEGLPAK GYDPGRDTYQ**  **551 APPADRSSVN VAVSPTSDRL QLLAGFEPWD GKDANGIPIL IKCQGKTTTD**  **601 HISMAGPWLK YRGHLDNISN NMLIGAVNAE NGKANSVKNK FTGEYDAVPA**  **651 TARDYKARGV KWVVIGDWNY GEGSSREHAA LEPRHLGGLA IITRSFARIH**  **701 ETNLKKQGML PLTFADPADY DKINPEDTVD LLCTQLEVGK PMTLRVHPKD**  **751 GSAPFDISLN HTFNESQIEW FKDGSALNTM ARKSGAK**  **63 - 73 458.9341 1373.7805 1373.7779 0.0026 1 R.RLQRPLTYAEK.V**  **74 - 89 636.6498 1906.9276 1906.9173 0.0102 0 K.VLYSHLDDPHGQEIER.G**  **148 - 165 662.0120 1983.0142 1982.9949 0.0193 1 R.ANEINKEVYDFLASATAK.Y**  **154 - 165 657.8387 1313.6628 1313.6503 0.0125 0 K.EVYDFLASATAK.Y**  **256 - 263 400.7673 799.5200 799.5167 0.0033 0 K.VAGLLTVK.G**  **417 - 429 717.8925 1433.7704 1433.7514 0.0190 0 K.SLFTVTPGSEQIR.A**  **512 - 520 538.3137 1074.6128 1074.6073 0.0055 1 K.FNPLTDKLK.D**  **521 - 540 709.3689 2125.0849 2125.0691 0.0157 1 K.DKDGNEFLLQPPTGEGLPAK.G**  **523 - 540 941.9929 1881.9712 1881.9472 0.0240 0 K.DGNEFLLQPPTGEGLPAK.G**  **541 - 556 593.6111 1777.8115 1777.8020 0.0095 1 K.GYDPGRDTYQAPPADR.S**  **557 - 569 659.8389 1317.6632 1317.6525 0.0108 0 R.SSVNVAVSPTSDR.L**  **583 - 592 527.3240 1052.6334 1052.6230 0.0105 0 K.DANGIPILIK.C)**  **639 - 653 547.2748 1638.8026 1638.8002 0.0024 1 K.NKFTGEYDAVPATAR.D**  **641 - 653 699.3455 1396.6764 1396.6623 0.0142 0 K.FTGEYDAVPATAR.D**  **677 - 684 461.7411 921.4676 921.4668 0.0009 0 R.EHAALEPR.H**  **685 - 694 525.8295 1049.6444 1049.6346 0.0099 0 R.HLGGLAIITR.S** |

***Footnote:*** ^1^Mass 1 = Molecular mass of the protein observed in Mascot search. ^2^Mascot score 2 = >40 indicate identification or extensive homology (p<0.05). ^3^Peptide matched 3 = Number of peptides matched with protein in MS/MS query. ^4^pI 4 = Isoeletric point of the protein observed in Mascot search.
